# Involvement of impaired carnitine-induced fatty acid oxidation in experimental and human diabetic kidney disease

**DOI:** 10.1172/jci.insight.179362

**Published:** 2025-05-22

**Authors:** Sakuya Ito, Kensei Taguchi, Goh Kodama, Saori Kubo, Tomofumi Moriyama, Yuya Yamashita, Yunosuke Yokota, Yosuke Nakayama, Yusuke Kaida, Masami Shinohara, Kyoko Tashiro, Keisuke Ohta, Sho-ichi Yamagishi, Kei Fukami

**Affiliations:** 1 Division of Nephrology, Department of Medicine, and; 2 Research Institute of Medical Mass Spectrometry, Kurume University School of Medicine, Kurume, Japan.; 3 Tokyo Animal & Diet Department, CLEA Japan, Inc., Osaka, Japan.; 4 Advanced Imaging Research Center, Kurume University School of Medicine, Kurume, Japan.; 5 Division of Diabetes, Metabolism, and Endocrinology, Department of Medicine, Showa University Graduate School of Medicine, Tokyo, Japan.

**Keywords:** Cell biology, Nephrology, Chronic kidney disease, Diabetes, Fatty acid oxidation

## Abstract

Diabetic kidney disease (DKD) is the leading cause of end-stage kidney disease. Kidney tubular cells have a high energy demand, dependent on fatty acid oxidation (FAO). Although carnitine is indispensable for FAO, the pathological role of carnitine deficiency in DKD is not fully understood. We showed here that ectopic lipid accumulation owing to impaired FAO increased in patients with DKD and inversely correlated with kidney function. Organic cation/carnitine transporter 2–deficient (OCTN2-deficient) mice exhibited systemic carnitine deficiency with increased renal lipid accumulation. Cell death and inflammation were induced in OCTN2-deficient, but not wild-type, tubular cells exposed to high salt and high glucose. Compared with Spontaneously Diabetic Torii (SDT) fatty rats, uninephrectomized SDT fatty rats fed with 0.3% NaCl showed higher lipid accumulation and increased urinary albumin excretion with kidney dysfunction and tubulointerstitial injury, all of which were ameliorated by l-carnitine supplementation via stimulating FAO and mitochondrial biogenesis. In our single-center randomized control trial with patients undergoing peritoneal dialysis, l-carnitine supplementation preserved residual renal function and increased urine volume, the latter of which was correlated with improvement of tubular injury. The present study demonstrates the pathological role of impairment of carnitine-induced FAO in DKD, suggesting that l-carnitine supplementation is a potent therapeutic strategy for this devastating disorder.

## Abstract 2

Diabetes in corporation with salt-sensitive hypertension induces carnitine deficiency accelerating kidney damage via mitochondrial dysfunction, which can be blocked by L-carnitine supplementation.

## Introduction

According to the International Diabetes Federation Diabetes Atlas 2021, approximately 537 million individuals were reported to have diabetes worldwide, the number of which is estimated to grow to over 783.2 million in the next 20 years (1). Among various diabetic complications, diabetic nephropathy is a leading cause of cardiovascular disease and associated with high mortality rate in patients with diabetes (2). In recent years, some diabetic patients with other comorbidities, such as hypertension, did not develop a typical clinical course of diabetic nephropathy. Thus, the broad concept, namely diabetic kidney disease (DKD), has been globally accepted, and the number of patients with DKD has been increasing (3). Traditional interventions, including intensive glucose control and strict blood pressure control, are straightforward to slow down the progression of DKD (4); however, because of the complex pathological mechanisms of DKD, a multipronged therapeutic approach is urgently required. Furthermore, it has been pointed out that rodent models of DKD show variable human translatability (5), which may be an impediment for creating new therapeutic options. Therefore, development of a novel DKD animal model is also actually desired.

The kidney is one of the most energy-consuming organs (6). Thus, kidney proximal tubular cells (PTCs) possess a large number of mitochondria, which occupy 30% of the cells’ volume to maintain the homeostasis in the kidneys (7). It is increasingly accepted that mitochondrial dysfunction causes progressive DKD (8). Among several pathological mechanisms caused by impaired function of mitochondria, defective fatty acid oxidation (FAO) may be a causative factor to advance DKD (9, 10). Indeed, mitochondrial FAO is a major metabolic pathway for fatty acid consumption and is essential for the cells not only to maintain energy homeostasis but also to regulate cell membrane stabilization (11) and cellular detoxification (12). However, key enzymes for FAO, including carnitine palmitoyltransferase 1A (CPT1a) and carnitine palmitoyltransferase 2 (CPT2), were downregulated, and peroxisome proliferator–activated receptor-α, a master regulator for FAO, was also reduced in the kidneys of patients with diabetes, all of whose levels were inversely correlated with kidney fibrosis (13). By contrast, CPT1a overexpression in the PTC has been reported to prevent kidney fibrosis in rodents (13, 14); thus, restoring the enzymes and transcriptional factor related to FAO is expected to be one of the major strategies to prevent the progression of DKD.

Carnitine is indispensable for the transfer of long-chain fatty acids across the inner mitochondrial membrane and subsequent β-oxidation in the mitochondria (15). Carnitine is known to be reabsorbed by PTCs through organic cation/carnitine transporter 2 (OCTN2) and then converted by CPT1a to acyl-carnitine (Acyl-C), which functions as a shuttle to transport long-chain fatty acids into the mitochondrial matrix (16). In a clinical study, total and free carnitine levels in the plasma were lower in diabetic patients with vascular complications when compared with those without complications (17), indicating that carnitine deficiency may be a potent contributor to impair mitochondrial FAO, thereby leading to the development and progression of diabetic vascular complications. Indeed, l-carnitine supplementation has been already applied to prevent dialysis-related complications only for patients with hemodialysis (18). Therefore, l-carnitine supplementation is anticipated to have a protective effect against progression of DKD; however, it has yet to be investigated (19, 20).

In the present study, we investigated the carnitine dynamics of human and experimental rodent models of DKD and further examined the pathological role of carnitine deficiency and resultantly impaired FAO in DKD. Also, we studied the protective effects of l-carnitine supplementation against DKD in both animal models and patients undergoing peritoneal dialysis (PD).

## Results

### Middle-to-long-chain Acyl-C and ectopic lipid accumulation are increased in patients with DKD.

**Figure 1:**
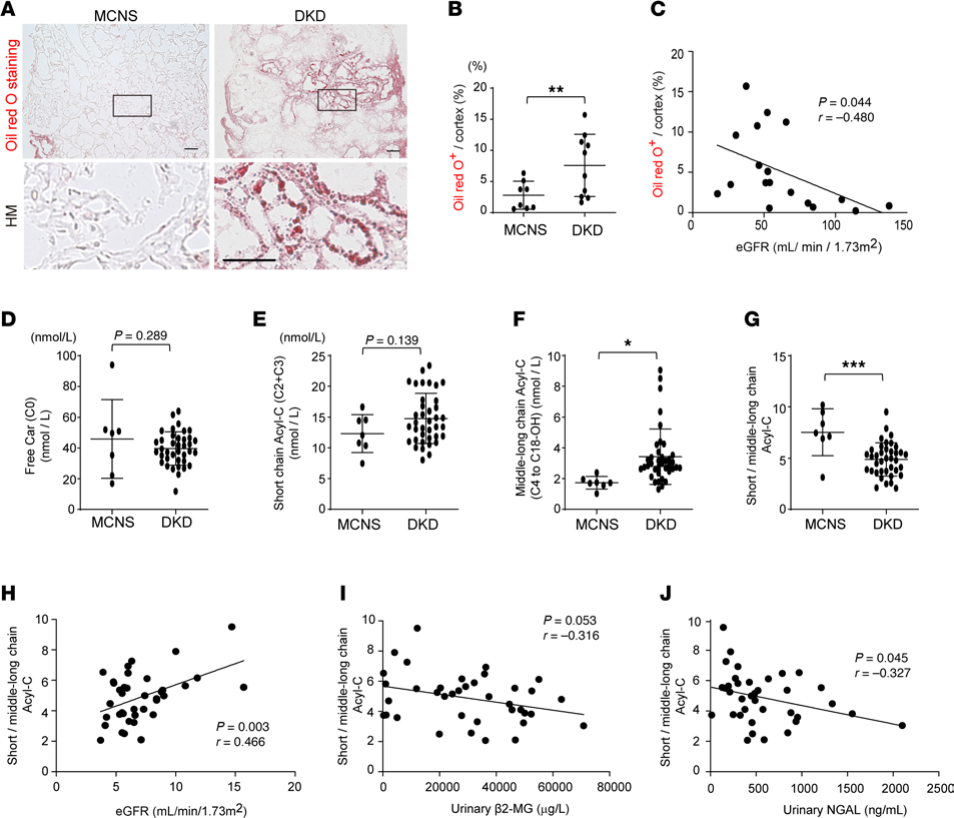
Middle- and long-chain Acyl-C and kidney ectopic fat accumulation are increased in patients with DKD. (**A**) Representative images of Oil Red O staining in kidneys of MCNS and DKD. Scale bar: 50 μm. (**B**) Corresponding quantification of Oil Red O^+^ area/cortex (%). MCNS, *n* = 8; DKD, *n* = 10. (**C**) Correlation of Oil Red O^+^ area/cortex (%) and eGFR (mL/min/1.73 m^2^). *n* = 19. (**D**) Plasma free Car (nmol/L), (**E**) plasma short-chain Acyl-C (nmol/L), (**F**) middle-to-long-chain Acyl-C (nmol/L), and (**G**) ratio of plasma short-chain Acyl-C to middle-to-long-chain Acyl-C in patients with MCNS (*n* = 7) and patients with DKD (*n* = 36). (**H**) Correlation between ratio of plasma short-chain Acyl-C to middle-to-long-chain Acyl-C and eGFR (mL/min/1.73 m^2^). (**I**) Correlation between ratio of plasma short-chain Acyl-C to middle-to-long-chain Acyl-C and urinary β2-MG (μg/L). (**J**) Correlation between ratio of plasma short-chain Acyl-C to middle-to-long-chain Acyl-C and urinary NGAL (ng/mL). Data are presented as means ± SD. Unpaired, 2-tailed Student’s *t* test (**B** and **D**–**G**) and Pearson’s correlation coefficient (**C** and **H**–**J**) were performed to determine *P* value. *P < 0.05, **P < 0.01, and ***P < 0.001. MCNS, minor change nephrotic syndrome; DKD, diabetic kidney disease; Car, carnitine; Acyl-C, acylcarnitine; eGFR, estimated glomerular filtration rate; β2-MG, β2-microglobulin; NGAL, neutrophil gelatinase-associated lipocalin.

First, to investigate whether ectopic lipid accumulation is promoted in the kidneys of patients with DKD, we performed Oil O Red staining with kidney biopsy specimens of patients with minimal change nephrotic syndrome (MCNS) and DKD. There was no significant difference in sex, age, proteinuria, serum creatinine, hemoglobin A1c, and serum triglycerides between the 2 groups. Hemoglobin value, estimated glomerular filtration rate (eGFR), high-density lipoprotein–cholesterol (HDL-cholesterol), and low-density lipoprotein–cholesterol (LDL-cholesterol) were lower in patients with DKD compared with those of patients with MCNS. The body mass index was lower in patients with MCNS compared with that of patients with DKD (Table 1). We observed there was more ectopic lipid accumulation in the kidneys of patients with DKD compared with those with MCNS (Figure 1, A and B). Also, the ectopic lipid accumulation was negatively correlated with eGFR (*r* = –0.480, *P* = 0.044; Figure 1C).

Next, to examine the carnitine profiles and kinetics in patients with DKD, we performed a prospective observational study at Kurume University Hospital. Regarding the clinical background, the patients with DKD displayed higher values of age, serum albumin, hemoglobin A1c, serum BUN, serum creatinine, and urinary β2-microglobulin (β2-MG) than those in patients with MCNS (Table 2). By contrast, patients with DKD had decreased hemoglobin, eGFR, HDL-cholesterol, LDL-cholesterol, and urinary NAG levels. However, urinary protein and serum triglyceride levels were similar between the DKD and MCNS groups (Table 2). Although there were no differences in free carnitine or short-chain Acyl-C between the 2 groups (Figure 1, D and E), middle-to-long-chain Acyl-C were higher in patients with DKD than those with MCNS (Figure 1F). Detailed information of carnitine profile is presented in Table 2. β-Oxidation is a catabolic process by which long-chain fatty acids are broken into free and short chains in the cytosol; thus, the decreased ratio of short-chain Acyl-C/middle-to-long-chain Acyl-C is considered a marker of impaired β-oxidation (21). The ratio of short-chain Acyl-C/middle-to-long-chain Acyl-C was lower in patients with DKD than those with MCNS (Figure 1G). Furthermore, eGFR was positively correlated with the ratio of short-chain Acyl-C/middle-to-long-chain Acyl-C in patients with DKD (*r* = 0.466, *P* = 0.003; Figure 1H). There was an inverse correlation between the ratio of short-chain Acyl-C/middle-to-long-chain Acyl-C and tubular injury markers, including urinary β2-MG (Figure 1I) and neutrophil gelatinase-associated lipocalin (NGAL) (Figure 1J), in patients with DKD.

### Carnitine deficiency–derived ectopic lipid accumulation induces tubular cell death and inflammation in combination with high salt and high glucose.

**Figure 2:**
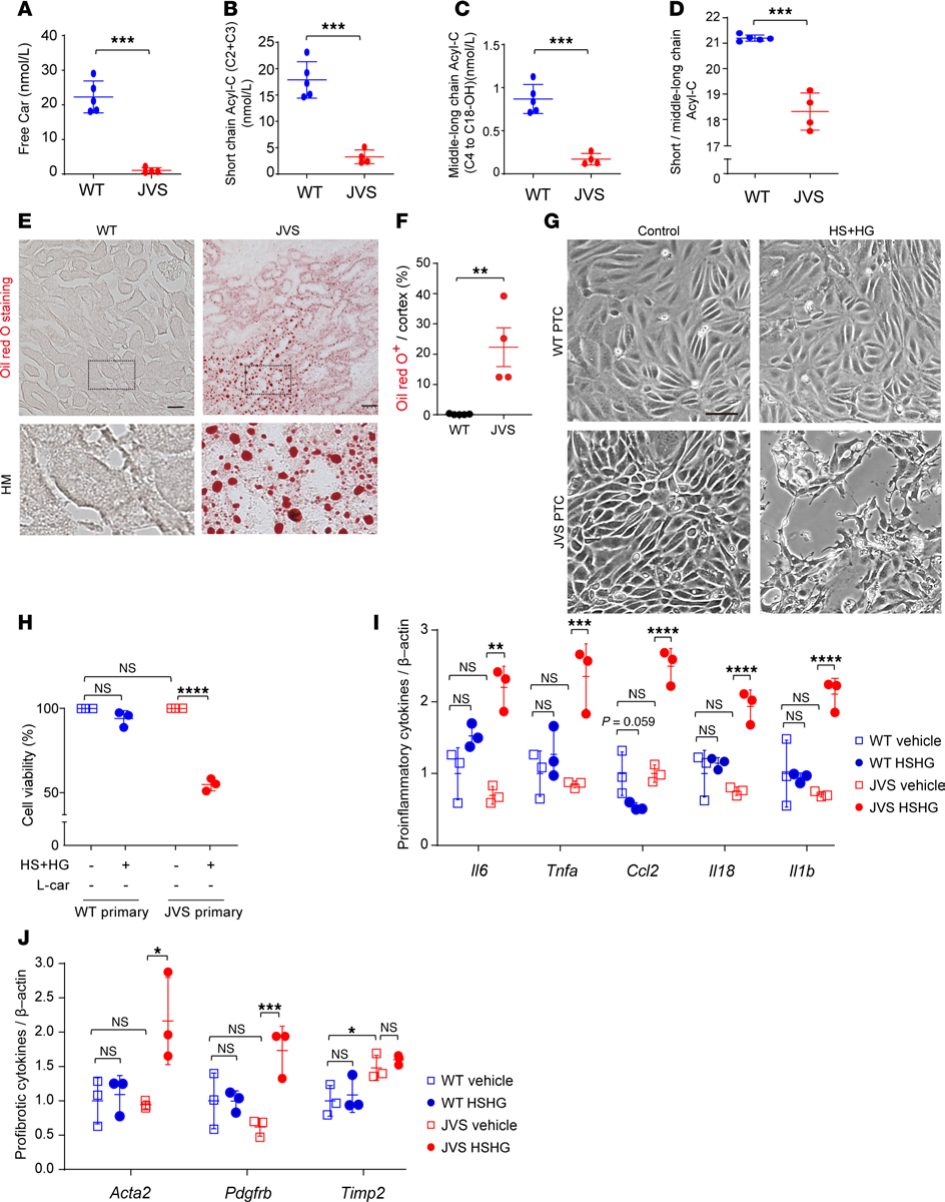
Carnitine deficiency drives ectopic fat accumulation in the kidneys. (**A**) Plasma free Car (nmol/L), (**B**) plasma short-chain Acyl-C (nmol/L), (**C**) plasma middle-to-long-chain Acyl-C (nmol/L), and (**D**) ratio of plasma short-chain Acyl-C to middle-to-long-chain Acyl-C in the control (*n* = 5) and JVS mice (*n* = 4). (**E**) Representative images of Oil Red O staining in WT and JVS mice. Scale bar: 50 μm. (**F**) Corresponding quantification of Oil Red O^+^ area/cortex (%). WT (*n* = 5) and JVS mice (*n* = 5). Scale bar: 50 μm. (**G**) Representative phase contrast images of primary PTCs obtained from WT and JVS mice in the presence or absence of HS+HG treatment. Scale bar: 20 μm. (**H**) The corresponding data of cell viability in **G**. *n* = 3, respectively. (**I**) Real-time PCR for pro-inflammatory cytokines in primary PTCs obtained from WT and JVS mice in the presence or absence of HS+HG treatment. *n* = 3, respectively. (**J**) Real-time PCR for pro-fibrotic markers. *n* = 3, respectively. Data are presented as means ± SD. Unpaired, 2-tailed Student’s *t* test (**A**–**D** and **F**) and 1-way ANOVA with Tukey’s post hoc test (**H**–**J**) were performed to determine *P* value. *P < 0.05, **P < 0.01, ***P < 0.001, and ****P < 0.0001. WT, wild-type; JVS, juvenile visceral steatosis; PTC, proximal tubular cell; HS, high salt; HG, high glucose; HM, high magnitude.

To investigate the causal relationship between carnitine deficiency and ectopic lipid accumulation, we employed juvenile visceral steatosis (JVS) mice, an animal model with primary carnitine deficiency caused by a mutation of the gene encoding OCTN2 (22). JVS mice exhibited various organ failure, such as fatty liver, cardiac hypertrophy, and growth retardation (23). Activity of OCTN2 in JVS mice declined by 64% compared with that of wild-type (WT) mice (22). Our liquid chromatography-tandem mass spectrometry (LC-MS/MS) revealed that plasma free carnitine, short-chain Acyl-C, and middle-to-long-chain Acyl-C were decreased in JVS mice compared with those in WT mice (Figure 2, A–C). The ratio of short-chain Acyl-C/middle-to-long-chain Acyl-C was reduced in JVS mice (Figure 2D and Supplemental Figure 1; supplemental material available online with this article; https://doi.org/10.1172/jci.insight.179362DS1). Furthermore, ectopic lipids massively accumulated in the kidneys of JVS mice compared with those of WT mice (Figure 2, E and F). To further examine the involvement of carnitine deficiency–evoked lipid accumulation in PTC injury, we cultured PTCs isolated from both WT and JVS mice and exposed them to high salt and high glucose (HS+HG) for 7 days. Under basal condition, there was no significant difference in cell viability of PTCs between the 2 groups. Although HS+HG treatment did not affect cell viability of PTCs isolated from WT mice, it significantly decreased viable cell number of PTCs derived from JVS mice (Figure 2, G and H). As is the case in viable cell number, gene expression levels of pro-inflammatory cytokines, including *Il6*, *Tnfa*, *Ccl2*, *Il18*, and *Il1b*, in the PTCs isolated from JVS mice were almost similar to those from WT mice under basal condition; however, these gene expressions were significantly increased by HS+HG treatment in PTCs isolated from JVS mice but not WT mice (Figure 2I). Similarly, gene expression levels of pro-fibrotic markers, such as *Acta* and *Pdgfrb*, were increased by the treatment of HS+HG only in PTCs derived from JVS mice (Figure 2J). On the other hand, there was a significant difference of *Timp2* levels, one of the markers of kidney injury (24), between the PTCs from JVS mice and WT mice; although their expression levels were not affected by HS+HG treatment in either group, they were higher in PTCs of JVS mice than those of WT mice (Figure 2J).

### High salt–loaded nephrectomized DKD rats develop carnitine deficiency and tubular injury along with ectopic lipid accumulation.

**Figure 3:**
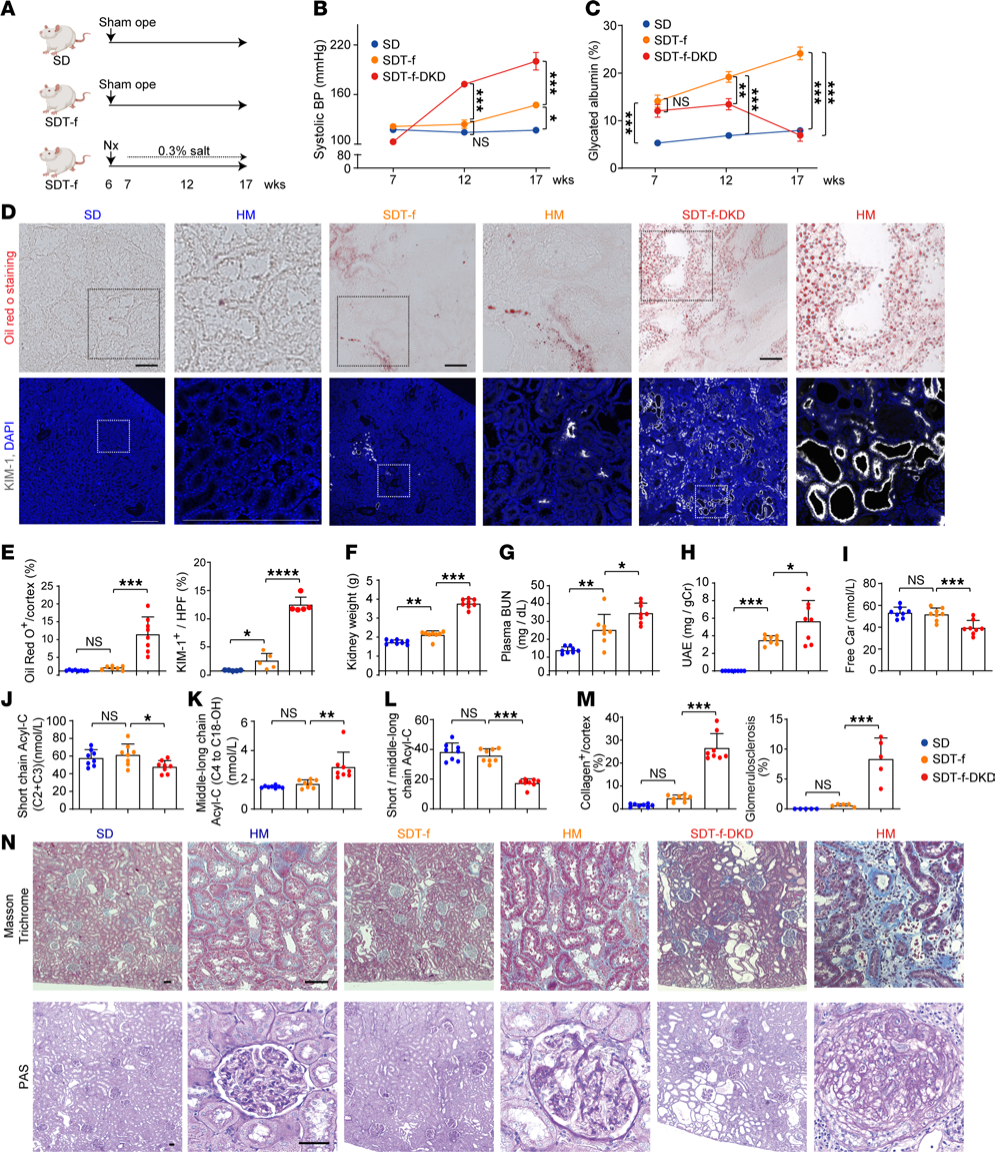
Clinical characteristics of SDT-f rats and DKD model rats. (**A**) Scheme of the experiment. (**B**) Line graph shows systolic BP (mmHg) and (**C**) plasma GA (%) at 7, 12, and 17 weeks in SD (*n* = 8), SDT-f (*n* = 8), and SDT-f-DKD (*n* = 8). (**D**) Representative images of Oil Red O staining (scale bar: 50 μm) and KIM-1–labeled kidneys of SD (*n* = 5), SDT-f (*n* = 5), and SDT-f-DKD (*n* = 5) at 17 weeks (scale bar: 500 μm). (**E**) The corresponding quantitation for Oil Red O^+^ area/cortex (%) and KIM-1^+^ area/HPF (%). (**F**) Kidney weight (g), (**G**) plasma BUN (mg/dL), and (**H**) urinary albumin excretion (mg/gCr) in SD (*n* = 8), SDT-f (*n* = 8), and SDT-f-DKD (*n* = 8) at 17 weeks. (**I**) Plasma free Car (nmol/L), (**J**) plasma short-chain Acyl-C (nmol/L), (**K**) plasma middle-to-long-chain Acyl-C (nmol/L), and (**L**) ratio of short-chain Acyl-C/middle-to-long-chain Acyl-C in SD (*n* = 8), SDT-f (*n* = 8), and SDT-f-DKD (*n* = 8) at 17 weeks. (**M**) Corresponding quantification of proportion of the sclerotic glomerulus (%) and collagen deposition area/cortex (%) in **N**. (**N**) Representative images of PAS and Masson’s trichrome staining in SD (*n* = 8), SDT-f (*n* = 8), and SDT-f-DKD (*n* = 8). Scale bar: 50 μm. Data are presented as means ± SD. One-way ANOVA with Tukey’s post hoc test (**B**, **C**, and **E**–**M**) were performed to determine *P* value. *P < 0.05, **P < 0.01, ***P < 0.001, and ****P < 0.0001. SDT, Spontaneously Diabetic Torii, GA, glycated albumin; KIM-1, kidney injury molecule-1; SDT-f, Spontaneously Diabetic Torii-fatty; SD, Sprague-Dawley; BP, blood pressure; Cr, creatinine; PAS, periodic acid–Schiff; HPF, high-power field; Nx, uninephrectomy; ope, operation.

Spontaneously Diabetic Torii Lepr fa (SDT-f) rats, established by introducing the *fa* allele of Zucker fatty rats into the SDT rat genome, is known to be a model of obese type 2 diabetes (25). While serum concentrations of glucose, triglycerides, and total cholesterol are gradually elevated over time, kidney impairment is mild even at 32 weeks of age in SDT-f rats (26). To establish a rat model of DKD with carnitine deficiency and kidney lipid accumulation, uninephrectomy was performed in SDT-f rats, which were then administered 0.3% salt–containing drinking water (27). The uninephrectomized SDT-f rats fed with high salt were named SDT-f-DKD rats (Figure 3A). Although systolic blood pressure (BP) was significantly higher in 17-week-old SDT-f rats than that of age-matched Sprague-Dawley (SD), it was further elevated in 12-week-old SDT-f-DKD rats compared with age-matched SDT-f rats, and the gap between the 2 groups widened further at 17 weeks of age (Figure 3B). While glycated albumin levels in plasma were significantly elevated in SDT-f rats compared with SD rats during the study periods, those of 12-week-old and 17-week-old SDT-f-DKD rats were significantly lower compared with age-matched SDT-f rats (Figure 3C).

Although compared with SD rats, SDT-f rats exhibited larger kidney weight; higher BUN, urinary albumin excretion (UAE), total cholesterol, triglycerides, HDL-cholesterol, and plasma insulin; and increased kidney injury molecule-1–positive (KIM-1^+^) cells, there were no significant differences in kidney lipid accumulation, plasma free carnitine, short-chain Acyl-C, middle-to-long-chain Acyl-C, ratio of short-chain Acyl-C/middle-to-long-chain Acyl-C, expression levels of collagen in the kidneys, or glomerulosclerosis between the 2 groups (Figure 3, D–N, and Supplemental Table 1). Kidney lipid accumulation, BUN, UAE, plasma middle-to-long-chain Acyl-C, KIM-1^+^ and collagen^+^ cells, glomerulosclerosis, plasma lipid parameters except for HDL-cholesterol, and glucagon levels were significantly increased in SDF-f-DKD rats compared with SDT-f rats, while plasma free carnitine, short-chain Acyl-C, and the ratio of short-chain Acyl-C/middle-to-long-chain Acyl-C were decreased in SDS-f-DKD rats (Figure 3, D–N, and Supplemental Table 1). Lipidomics results showed accumulated lipids consisted of mainly triglycerides (Supplemental Figure 2). Detailed information in terms of plasma and urinary carnitine profiles in SDT-f and SDT-f-DKD rats is shown in Supplemental Figure 3.

### FAO-related enzymes and peroxisome proliferator–activated receptor-γ coactivator 1α are reduced in SDT-f-DKD rats.

**Figure 4:**
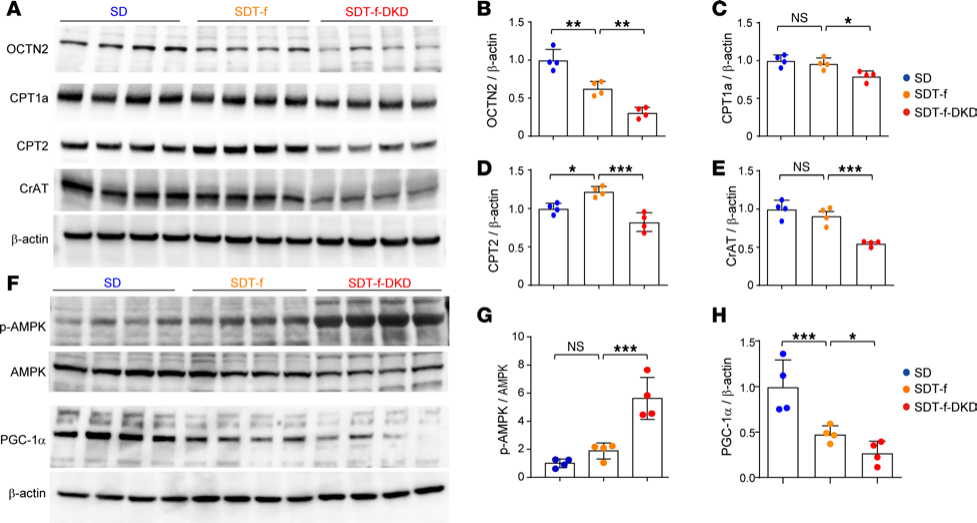
FAO-related transporter and enzymes are reduced in SDT-f-DKD rats. (**A**) Western blots for OCTN2, CPT1a, CPT2, CrAT, and β-actin in the kidneys of SD, SDT-f, and SDT-f-DKD rats. (**B**) Quantification of OCTN2/β-actin, (**C**) CPT1a/β-actin, (**D**) CPT2/β-actin, and (**E**) CrAT/β-actin in SD (*n* = 4), SDT-f (*n* = 4), and SDT-f-DKD rats (*n* = 4). (**F**) Western blots for p-AMPK, AMPK, PGC-1α, and β-actin in SD, SDT-f, and SDT-f-DKD rats. (**G**) Ratio of p-AMPK/AMPK and (**H**) quantification of PGC-1α /β-actin in SD (*n* = 4), SDT-f (*n* = 4), and SDT-f-DKD rats (*n* = 4). Data are presented as means ± SD. One-way ANOVA with Tukey’s post hoc test (**B**–**E**, **G**, and **H**) were performed to determine *P* value. *P < 0.05, **P < 0.01, and ***P < 0.001. OCTN2, organic cation transporter 2; CPT1a, carnitine palmitoyltransferase 1a; CPT2, carnitine palmitoyltransferase 2; CrAT, carnitine acetyltransferase; p-AMPK, phosphorylated AMP-activated protein kinase; PGC-1α, peroxisome proliferator–activated receptor-γ coactivator-1α.

We then evaluated the expression levels of OCTN2 and key enzymes of FAO, such as CPT1a, CPT2, and carnitine acetyltransferase (CrAT), in SD, SDT-f, and SDT-f-DKD rats. OCTN2 was reduced in SDT-f rats compared with SD rats, which was further decreased in SDT-f-DKD rats having carnitine deficiency (Figure 4, A and B). Among FAO-related enzymes, only CPT2 protein expression was increased in SDT-f rats compared with SD rats, whereas CPT1a, CPT2, and CrAT were significantly lower in SDT-f-DKD rats than those of SDT-f rats (Figure 4, A and C–E). Furthermore, although there was no significant difference of phosphorylated AMP-activated protein kinase (p-AMPK) levels between SDT-f rats and SD rats (Figure 4, F and G), peroxisome proliferator–activated receptor-γ coactivator 1α (PGC-1α), a transcription coactivator that mainly regulates mitochondrial biogenesis (28), was significantly lower in SDT-f rats than SD rats (Figure 4, F and H). Compared with SDT-f rats, p-AMPK levels were increased, while PGC-1α was further decreased in SDT-f-DKD rats (Figure 4, F–H).

### Reduced FAO enzymes and morphological changes in mitochondria following the decline in OCTN2 occur in SDT-f-DKD rats.

**Figure 5:**
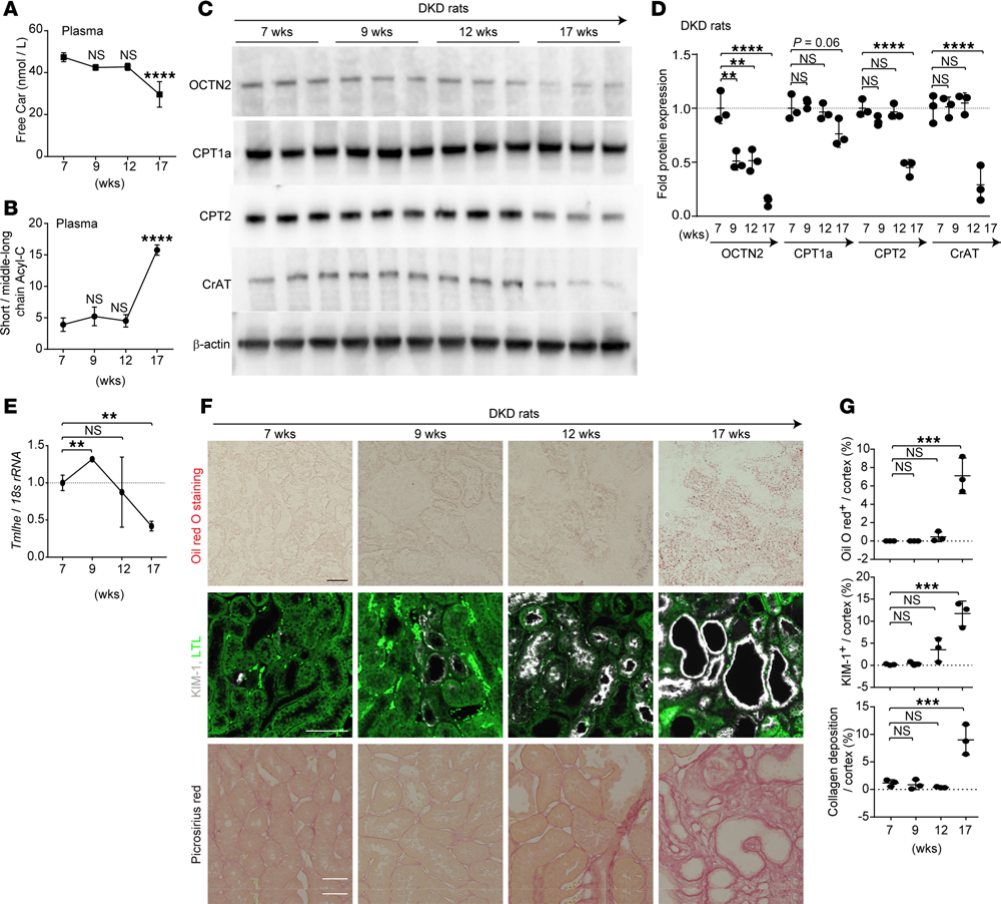
Reduced FAO enzymes and morphological changes in mitochondria following the decline in OCTN2 occur in SDT-f-DKD rats. (**A**) Plasma free Car (nmol/L) and (**B**) ratio of short-chain Acyl-C/middle-to-long-chain Acyl-C over the time course of SDT-f-DKD. (**C**) Western blots for OCTN2, CPT1a, CPT2, CrAT, and β-actin in the kidneys of SDT-f-DKD rats and (**D**) corresponding quantitation over the time course of SDT-f-DKD. *n* = 4, respectively. (**E**) Real-time PCR for *Tmlhe* in the kidneys of SDT-f-DKD rats at 7, 9, 12, and 17 weeks. *n* = 4, respectively. (**F**) Representative images of Oil Red O staining, immunofluorescence staining for KIM-1, and Picrosirius red staining over the time course of SDT-f-DKD and (**G**) the corresponding quantitation for Oil Red O^+^ area/cortex (%), KIM-1^+^/cortex (%), and collagen deposition area/cortex (%). Scale bar: 50 μm. *n* = 4, respectively. Data are presented as means ± SD. One-way ANOVA with Tukey’s post hoc test (**A**, **B**, **D**, **E**, and **G**) were performed to determine *P* value. **P < 0.01, ***P < 0.001, and ****P < 0.0001. *Tmlhe*, trimethyllysine hydroxylase, epsilon; wks, weeks; LTL, lotus tetragonolobus lectin.

**Figure 6:**
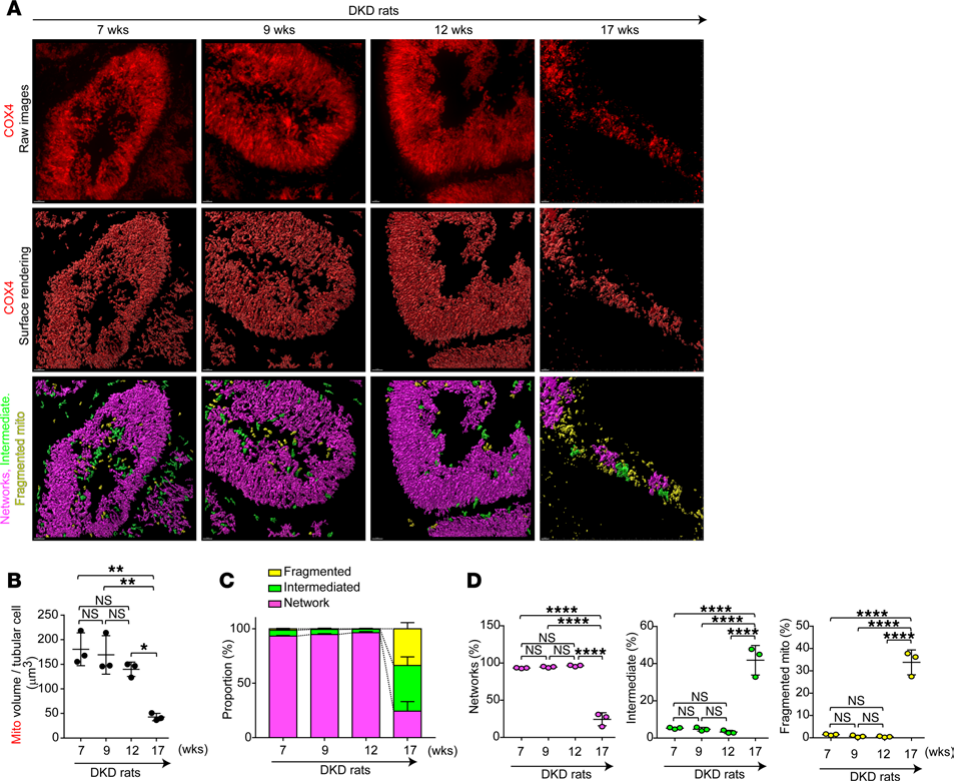
Mitochondrial fragmentation is observed in the late stage of disease progression in SDT-f-DKD rats. (**A**) Representative maximum intensity projections (top panels) and surface renderings (middle panels) of kidney tubular cells stained for COX IV (red). Bottom panels show surface renderings color coded for network morphology on the basis of sphericity: fragmented, yellow; intermediate, green; and filamentous, magenta. Scale bar, 2 μm. (**B**) Quantification of mitochondrial volume from surface renderings in **A**. *n* = 4, respectively. (**C**) Distribution of mitochondrial morphology on the basis of sphericity, presented as percentage volume of filamentous, fragmented, or intermediate mitochondria over the time course of SDT-f-DKD. (**D**) Quantification of mitochondrial morphology on the basis of sphericity. Data are presented as means ± SD. One-way ANOVA with Tukey’s post hoc test (**B** and **D**) were performed to determine *P* value. **P < 0.01, and ****P < 0.0001. COX, cytochrome *c* oxygenase.

To investigate the underlying cause of carnitine deficiency in SDT-f-DKD rats, we analyzed the carnitine profile in plasma; the expression levels of FAO enzymes and trimethyl lysine hydroxylase, epsilon (*Tmlhe*), a key biosynthesis enzyme of carnitine; and mitochondrial morphology at different time points in SDT-f-DKD rats. A significant reduction in free carnitine levels and the ratio of short-chain Acyl-C/middle-to-long-chain Acyl-C was first observed at 17 weeks of age (Figure 5, A and B). OCTN2 expression declined as early as 9 weeks of age, whereas the expression of FAO enzymes, including CPT1a, CPT2, and CrAT, was markedly reduced for the first time at 17 weeks of age (Figure 5, C and D). Gene expression levels of *Tmlhe* were initially upregulated at 9 weeks but showed a significant decline at 17 weeks compared with 7 weeks of age (Figure 5E). Ectopic lipid accumulation, KIM-1^+^ tubules, and kidney fibrosis were also first observed at 17 weeks of age (Figure 5, F and G). Furthermore, spinning disk super-resolution microscopy revealed PTC mitochondrial fragmentation with a significant reduction in total mitochondrial volume at 17 weeks of age (Figure 6, A and B). Surface rendering analysis showed that mitochondria formed networks under the normal condition (7), which was clearly observed (shown in magenta) in PTCs of SDT-f-DKD rats until 12 weeks of age, whereas the networks were broken into smaller mitochondrial fragments (shown in yellow) at 17 weeks of age (Figure 6, C and D). Detailed carnitine profiles and clinical characteristics in time course samples of SDT-f-DKD rats are shown in Supplemental Figure 4 and Supplemental Table 2, respectively.

### Supplementation with l-carnitine attenuates kidney dysfunction of SDT-f-DKD rats with normalizing carnitine profiles.

**Figure 7:**
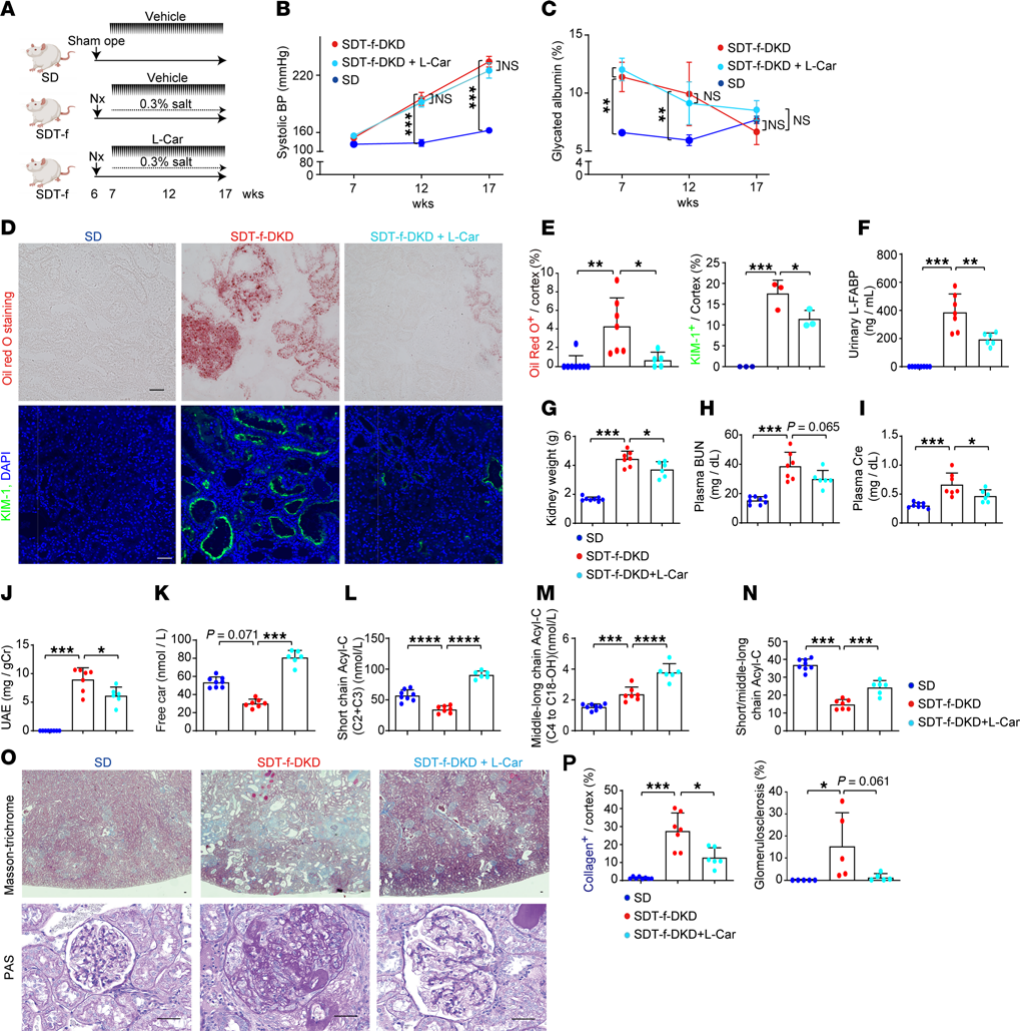
Supplementation with l-carnitine attenuates kidney injury in SDT-f-DKD rats. (**A**) Scheme of the experiment on SDT-f-DKD rats treated with l-carnitine. (**B**) Line graph shows systolic BP and (**C**) plasma GA value in SD (*n* = 8), SDT-f-DKD (*n* = 7), and SDT-f-DKD + L-car (*n* = 6) at 7, 12, and 17 weeks. (**D**) Representative images of Oil Red O staining and immunofluorescence staining for KIM-1. Scale bar: 50 μm. (**E**) Corresponding quantitation for Oil Red O^+^ area/cortex (%) and KIM-1^+^ area/cortex in SD (*n* = 3–8), SDT-f-DKD (*n* = 3–7), and SDT-f-DKD + L-car (*n* = 3–6) at 17 weeks. (**F**) Urinary L-FABP in SD (*n* = 8), SDT-f-DKD (*n* = 7), and SDT-f-DKD + L-car (*n* = 6). (**G**) Kidney weight (g), (**H**) plasma BUN, (**I**) plasma Cre, and (**J**) UAE in SD (*n* = 8), SDT-f-DKD (*n* = 7), and SDT-f-DKD + L-car (*n* = 6) at 17 weeks. (**K**) Plasma free Car (nmol/L), (**L**) short chain Acyl-C (C2+C3), (**M**) middle-to-long chain Acyl-C (C4 to C18-OH), and (**N**) ratio of short-chain Acyl-C/middle-to-long-chain Acyl-C in SD (*n* = 8), SDT-f-DKD (*n* = 7), and SDT-f-DKD + L-car (*n* = 6) at 17 weeks. (**O**) Representative images of Masson’s trichrome and PAS staining in the kidneys of SD, SDT-f-DKD, and SDT-f-DKD+L-car. Scale bar: 50 μm. (**P**) Corresponding quantification of collagen deposition area/cortex (%) and the percentage of glomerulosclerosis (%). Data are presented as means ± SD. One-way ANOVA with Tukey’s post hoc test (**B**, **C**, **E**–**N**, and **P**) were performed to determine *P* value. *P < 0.05, **P < 0.01, ***P < 0.001, and ****P < 0.0001. Cre, creatinine; L-FABP, liver type-fatty acid binding protein; UAE, urinary albumin excretion.

To examine the pathological role of impairment of carnitine-induced FAO and subsequent kidney lipid accumulation in our DKD model, SDT-f-DKD rats were treated with l-carnitine supplementation for 10 weeks (Figure 7A). Although l-carnitine supplementation did not affect systolic BP, glycated albumin, lipid parameters, body weight, or plasma insulin levels in SDT-f-DKD rats (Figure 7, B and C, and Supplemental Table 3), it significantly inhibited the increase in ectopic lipid accumulation (Figure 7, D and E), KIM-1^+^ PTCs (Figure 7, D and E), urinary liver type-fatty acid binding protein (L-FABP) (Figure 7F), kidney weight (Figure 7G), BUN (Figure 7H), plasma creatinine (Figure 7I), plasma level of glucagon (Supplemental Table 3), and UAE (Figure 7J). LC-MS/MS revealed that decreased plasma free carnitine (Figure 7K) and short-chain Acyl-C (Figure 7L) and increased middle-to-long-chain Acyl-C (Figure 7M) associated with the reduced ratio of short-chain Acyl-C/middle-to-long-chain Acyl-C (Figure 7N) were significantly ameliorated by l-carnitine supplementation in SDT-f-DKD rats except for plasma middle-to-long-chain Acyl-C levels. The increase in collagen deposition and glomerulosclerosis in SDT-f-DKD rats were also attenuated by l-carnitine supplementation (Figure 7, O and P). Detailed carnitine profiles in SDT-f-DKD rats with or without l-carnitine supplementation are shown in Supplemental Figure 5.

### Effects of l-carnitine supplementation on FAO-related enzymes and PGC-1α in SDT-f-DKD rats.

**Figure 8:**
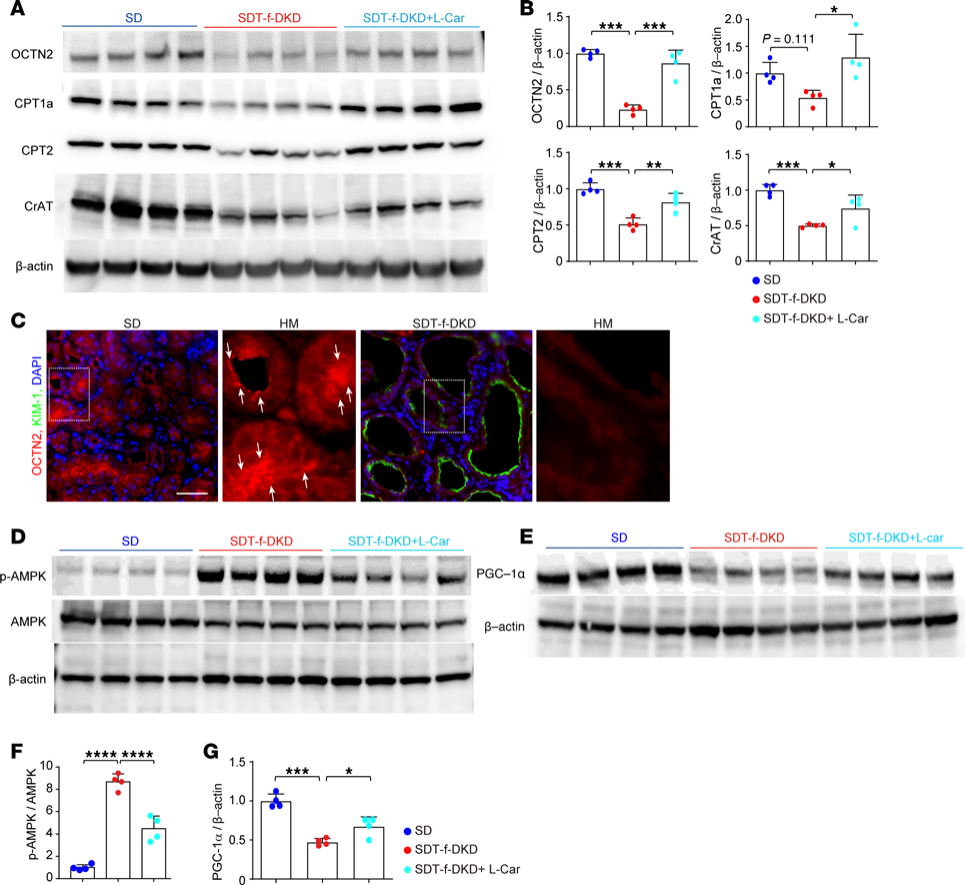
Carnitine-related transporters and enzymes are preserved by l-carnitine supplementation. (**A**) Representative Western blot images of carnitine-related transporters, such as OCTN2, CPT1a, CPT2, CrAT, and β-actin. (**B**) Quantification of OCTN2/β-actin, CPT1a/β-actin, CPT2/β-actin, and CrAT/β-actin. SD, *n* = 4; SDT-f-DKD, *n* = 4; SDT-f-DKD + L-car, *n* = 4. (**C**) Colocalization image of KIM-1 and OCTN2 in the cortex. The arrows indicate OCTN2 expression localized to the lumen of kidney tubular cells. Scale bar: 50 μm. (**D**) Representative Western blot images of p-AMPK, AMPK, (**E**) PGC-1α, and β-actin. (**F**) The ratio of p-AMPK/AMPK and (**G**) the ratio of PGC-1α/β-actin in SD (*n* = 4), SDT-f-DKD (*n* = 4), and SDT-f-DKD+L-car (*n* = 4). Data are presented as means ± SD. One-way ANOVA with Tukey’s post hoc test (**B**, **F**, and **G**) were performed to determine *P* value. *P < 0.05, **P < 0.01, ***P < 0.001, and ****P < 0.0001.

Western blotting analyses revealed that decreased renal levels of OCTN2, CPT1a, CPT2, and CrAT were significantly restored by the treatment with l-carnitine in SDT-f-DKD rats (Figure 8, A and B). Immunofluorescence staining showed that OCTN2 was localized in apical proximal tubules, whose expression levels were drastically suppressed in KIM-1^+^ PTCs (Figure 8C). Supplementation with l-carnitine supplementation significantly inhibited the increase in p-AMPK as well as the decrease in PGC-1α in SDT-f-DKD rats (Figure 8, D–G).

### Protective effects of l-carnitine supplementation on morphology and function of mitochondria in SDT-f-DKD rats.

**Figure 9:**
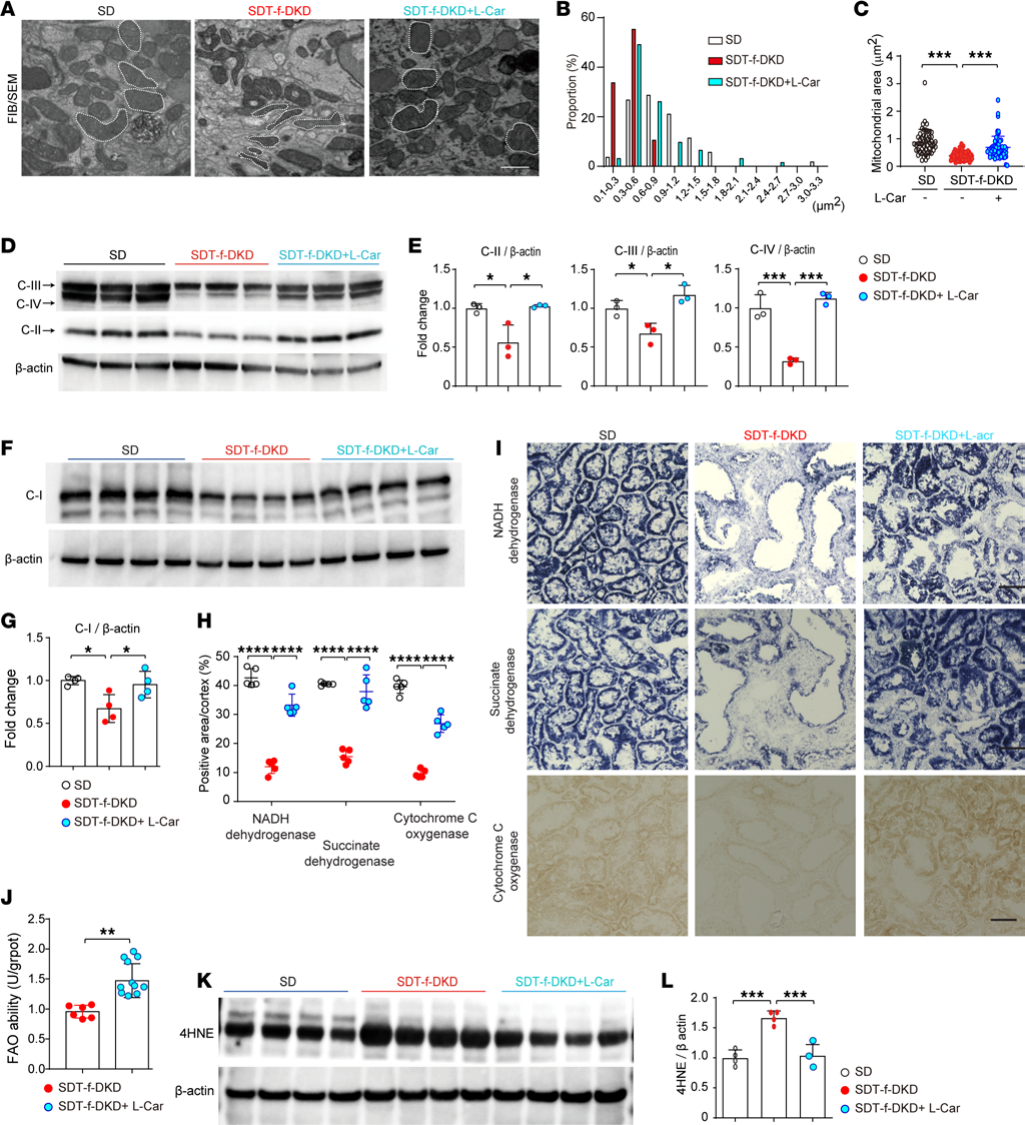
Carnitine supplementation restores mitochondria via PGC-1α and reduces oxidative stress in SDT-f-DKD rats. (**A**) Representative FIB/SEM images in the tubules of SD, SDT-f-DKD, and SDT-f-DKD + L-car. Scale bar: 500 nm. (**B**) Histogram of the mitochondrial area and (**C**) average area of mitochondria in SD (*n* = 52), SDT-f-DKD (*n* = 56), and SDT-f-DKD+L-car (*n* = 61) in Figure 7A. (**D**) Western blot images of C-II, C-III, and C-IV and (**E**) the corresponding quantitation for C-II, C-III, and C-IV/β-actin. SD, *n* = 3; SDT-f-DKD, *n* = 3; SDT-f-DKD+L-car, *n* = 3. (**F**) Western blot image of mitochondrial respiratory complex I and (**G**) the corresponding quantitation of the mitochondrial respiratory complex I/β-actin. SD, *n* = 4; SDT-f-DKD, *n* = 4; SDT-f-DKD+L-car, *n* = 4. (**H**) Corresponding quantitation for positive area/cortex (%) in **I**. (**I**) Representative images for NADH dehydrogenase, succinate dehydrogenase, and cytochrome *c* oxygenase in the kidneys of SD, SDT-f-DKD, and SDT-f-DKD+L-car rats. Scale bar: 50 μm. (**J**) FAO rate with kidney samples of SDT-f-DKD rats with or without L-car. (**K**) Western blot images of 4HNE in the kidneys of SD (*n* = 4), SDT-f-DKD (*n* = 4), and SDT-f-DKD+L-car (*n* = 4) and (**L**) the corresponding quantitation of 4HNE/β-actin. Data are presented as means ± SD. Unpaired, 2-tailed Student’s *t* test (**J**) and 1-way ANOVA with Tukey’s post hoc test (**C**, **E**, **G**, **H**, and **L**) were performed to determine *P* value. *P < 0.05, **P < 0.01, ***P < 0.001, and ****P < 0.0001. FIB/SEM, focused ion beam/scanning electron microscopes; 4HNE, 4-hydroxy-2-nonenal; C, mitochondrial respiratory complex.

We next investigated the effects of l-carnitine supplementation on mitochondrial morphology, respiratory function, and oxidative stress in SDT-f-DKD rats. Electron microscopy (EM) demonstrated that size of PTC mitochondria and their area were reduced in SDT-f-DKD rats compared with SD rats, which were restored by l-carnitine supplementation (Figure 9, A–C). Furthermore, damaged mitochondria displaying a ring/doughnut shape (29) were frequently observed in SDT-f-DKD rats, which were also improved by l-carnitine supplementation (Figure 9A). Supplementation with l-carnitine significantly restored the decreased representative protein levels of electron transport chain complexes I, II, III, and IV (Figure 9, D–G). Next, we analyzed the activity of electron transport chain complexes using fresh-frozen sections and found NADH dehydrogenase (complex I), succinate dehydrogenase (SDH) (complex II), and cytochrome *c* oxidase (complex IV) exhibited decreased activity staining in the kidneys of SDT-f-DKD rats, all of which were attenuated by l-carnitine supplementation (Figure 9, H and I). FAO rate with kidney lysates of SDT-f-DKD rats was significantly enhanced by l-carnitine supplementation (Figure 9J). Furthermore, l-carnitine inhibited the increased renal 4-hydroxy-2-nonenal (4HNE), a lipid peroxidation marker (30) (Figure 9, K and L).

### Salt-sensitive hypertension partially induces PTC carnitine deficiency with reduced plasma free carnitine.

**Figure 10:**
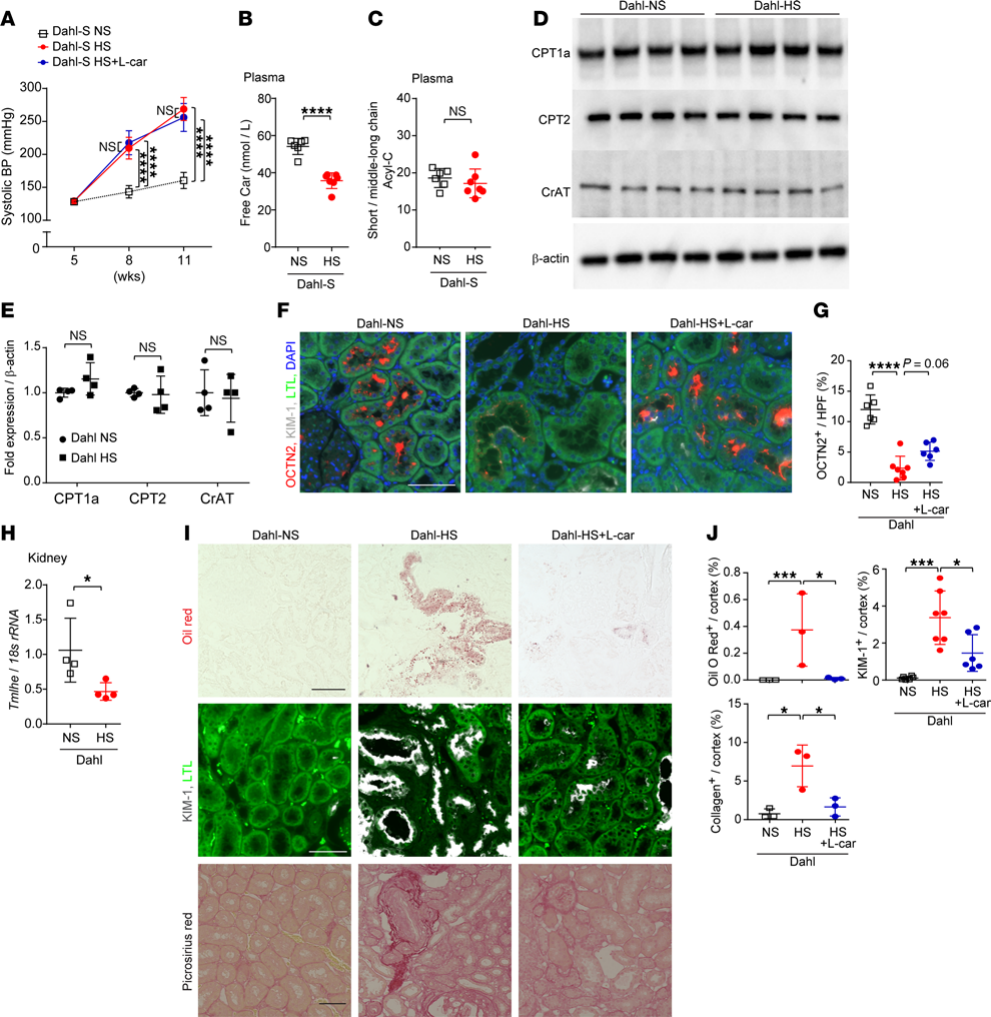
Salt-sensitive hypertension partially induces PTC carnitine deficiency with reduced plasma free Car. (**A**) Line graph shows systolic BP (mmHg) over time in Dahl-NS (*n* = 6), Dahl-HS (*n* = 7), and Dahl-HS+L-car (*n* = 6). (**B**) Plasma free Car (nmol/L) and (**C**) ratio of short-chain Acyl-C/middle-to-long-chain Acyl-C. (**D**) Western blots for CPT1a, CPT2, CrAT, and β-actin in the kidneys of Dahl-NS and Dahl-HS and (**E**) corresponding quantitation. *n* = 4, respectively. (**F**) Representative images for OCTN2 and (**G**) the corresponding quantitation for OCTN2^+^/HPF (%) in Dahl-NS (*n* = 6), Dahl-HS (*n* = 7), and Dahl-HS+L-car (*n* = 6). Scale bar, 50 μm. (**H**) Real-time PCR for *Tmlhe* in the kidneys of Dahl-NS and Dahl-HS. *n* = 4, respectively. (**I**) Representative images of Oil Red O staining, immunofluorescence staining for KIM-1, and Picrosirius red staining in Dahl-NS, Dahl-HS, and Dahl-HS+L-car. Scale bar, 50 μm. (**J**) The corresponding quantitation for Oil Red O^+^ area/cortex (%), KIM-1^+^/cortex (%), and collagen^+^ area/cortex (%). *n* = 4, respectively. Data are presented as means ± SD. Unpaired, 2-tailed Student’s *t* test (**B**, **C**, **E**, and **H**) and 1-way ANOVA with Tukey’s post hoc test (**G** and **J**) were performed to determine *P* value. *P < 0.05, ***P < 0.001, ****P < 0.0001. NS, normal salt.

To investigate whether salt-sensitive hypertension alone is implicated in carnitine deficiency, Dahl salt-sensitive rats were fed an 8% sodium diet, which increased systolic BP to 250 mmHg (Dahl-HS) (Figure 10A). Since Dahl rats remain normotensive when given a normal sodium diet, those fed with normal chow were used as the control group (Dahl-NS). We observed plasma free carnitine levels were reduced in Dahl-HS rats when compared with Dahl-NS rats at 11 weeks of age (Figure 10B), whereas the ratio of short-chain Acyl-C/middle-to-long-chain Acyl-C remained unchanged (Figure 10C). CPT1a, CPT2, and CrAT expression levels were not altered in Dahl-HS rats when compared with Dahl-NS rats at 11 weeks of age (Figure 10, D and E). However, OCTN2^+^ area/cortex (%) was reduced in Dahl-HS rats compared with Dahl-NS rats (Figure 10, F and G), and the decline was especially severe in KIM-1^+^ tubules. Furthermore, gene expression of *Tmlhe* was reduced in Dahl-HS compared with Dahl-NS at 11 weeks of age (Figure 10H). Although carnitine-induced FAO was not impaired as much as that in SDT-f-DKD rats, l-carnitine supplementation for 6 weeks had protective effects on ectopic lipid accumulation, tubular injury, and kidney fibrosis without affecting systolic BP (Figure 10, A, I, and J), suggesting that salt-sensitive hypertension partially induces carnitine deficiency possibly via reduction in reabsorption and biosynthesis of carnitine. Detailed carnitine profiles and clinical characteristics are shown in Supplemental Figure 6 and Supplemental Table 4, respectively.

### Supplementation with l-carnitine halts the decline in kidney function in patients with PD.

**Figure 11:**
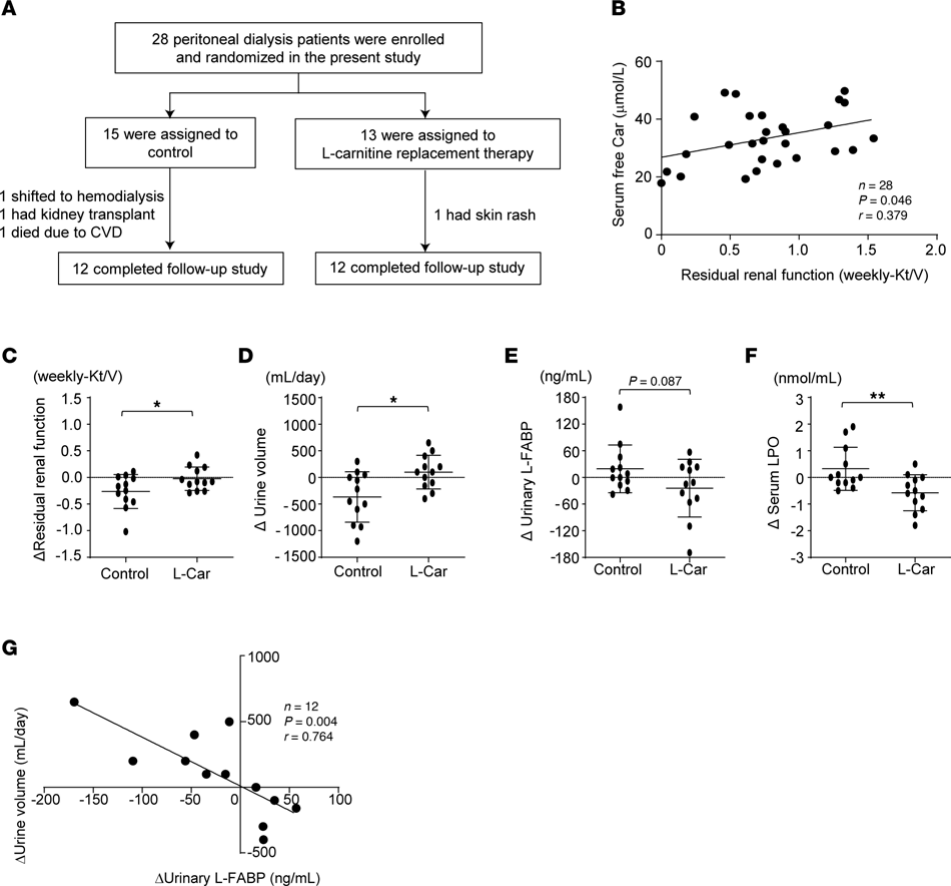
Supplementation with l-carnitine delays the progression of kidney injury in patients undergoing PD. (**A**) Study design. (**B**) Correlation RRF (weekly-Kt/V) and plasma free Car (nmol/L) at the beginning of the clinical study. *n* = 28, *P* = 0.046, *r* = 0.379. (**C**) ΔRRF, (**D**) Δurine volume, (**E**) Δurine L-FABP, and (**F**) Δserum LPO in the control (*n* = 12) and L-car–treated groups (*n* = 12) after 6 months of supplementation with L-car. (**G**) Correlation of Δurine volume and Δurine L-FABP in L-car–treated group (*n* = 12). *P =* 0.004, *r* = 0.764. Data are presented as means ± SD. Unpaired, 2-tailed Student’s *t* test (**C**–**F**) and Pearson’s correlation coefficient (**B** and **G**) were performed to determine *P* value. *P < 0.05, and **P < 0.01. RRF, residual renal function; LPO, lipid peroxidation.

To investigate whether l-carnitine supplementation could inhibit the progression of kidney injury in humans, we constructed a prospective, single-center, randomized control trial with patients undergoing PD. A total of 28 patients with PD were randomly assigned to 2 groups as follows: control group (*n* = 15) and oral l-carnitine treatment group (*n* = 13). Among them, 12 patients completed the 6-month l-carnitine treatment (Figure 11A). There was a significant correlation of serum free carnitine level with residual renal function (RRF) in PD patients (Figure 11B). At baseline, although body mass index was larger and number of diabetic patients was higher in the l-carnitine treatment group than the control, the other clinical and biochemical parameters did not differ between the 2 groups. Furthermore, there was no statistically significant difference in dialysis efficiency parameters at baseline except for dialysate volume between the groups; baseline dialysate volume was larger in the l-carnitine treatment group compared with control group. After a 6-month intervention, all the parameters except for plasma level of BUN did not change in either l-carnitine treatment or control group; BUN level was elevated after 6 months in control group but not l-carnitine–treated group (Supplemental Table 5). However, there were significant differences of the changes from baseline after 6-month intervention in RRF (ΔRRF), urine volume (Δurine volume), and serum lipid peroxidation (Δserum LPO) between the groups; compared with control group, ΔRRF and Δurine volume were significantly higher in l-carnitine treatment group, the latter of which was inversely correlated with urinary L-FABP, while Δserum LPO was significantly lower in the l-carnitine treatment group (Supplemental Table 5 and Figure 11, C–G).

At baseline, there were no statistically significant differences in carnitine levels at the baseline between the 2 groups except for C8, C10:1, C14, and C18:1-OH (Supplemental Table 6). l-Carnitine for 6 months significantly increased serum free carnitine (C0), short-chain Acyl-C (C2 to C3), middle-to-long-chain Acyl-C (C4 to C18-OH), and the ratio of short-chain Acyl-C/middle-to-long-chain Acyl-C (Supplemental Table 6).

## Discussion

The salient findings of this study are summarized as follows: (a) patients with DKD showed ectopic lipid accumulation in the kidneys and impairment of carnitine-induced FAO, both of which were linked to tubular injury; (b) carnitine deficiency combined with HS+HG exposure decreased viable cell number of PTCs in association with upregulation of pro-fibrotic and pro-inflammatory cytokines; (c) the decline in OCTN2 followed by inhibition of carnitine biosynthesis induced carnitine deficiency, leading to impairment of FAO and disruption of mitochondrial morphology in uninephrectomized type 2 diabetic rats with high salt loading; (d) carnitine deficiency–induced kidney dysfunction, tubular injury, and kidney fibrosis were attenuated by l-carnitine supplementation; (e) impaired carnitine-induced FAO disrupted mitochondrial biogenesis and morphology, resulting in oxidative stress generation in DKD rats, which were ameliorated by treatment with l-carnitine; (f) l-carnitine supplementation reduced oxidative stress and inhibited the progression of kidney damage in patients with PD partly by suppressing tubular injury.

We here demonstrated that patients with DKD displayed more ectopic lipid accumulation in the kidneys when compared with those in patients with MCNS. Lipid accumulation is commonly observed in patients with DKD (31), which is supposed to be due to increased uptake of fatty acid through CD36 (32) and KIM-1 (33), enhanced fatty acid synthesis, and downregulation of FAO genes; however, this is the first report to our knowledge demonstrating that carnitine deficiency–induced impairment of FAO was associated with the decline in eGFR and tubular injury along with the increased ectopic lipid accumulation in the kidneys of patients with DKD. Using an enzyme cycling method, we have previously reported that Acyl-C accumulates and the ratio of free carnitine/Acyl-C is decreased only in stage 5 chronic kidney disease (CKD) patients without diabetes (34). Meanwhile, eGFR of our DKD patients with ectopic lipid accumulation in the kidneys was 49.4 ± 25.3 mL/min/1.73 m^2^ (CKD stages 2–4) (Figure 1, A and B, and Table 1), suggesting that carnitine homeostasis would be more disrupted in diabetic conditions than nondiabetic circumstances, which could lead to more severe lipid accumulation and kidney injury. These findings indicate that abnormal carnitine homeostasis might be a potent biomarker to detect a high-risk population for end-stage kidney disease and a therapeutic target for the progression of DKD.

To verify whether abnormal carnitine homeostasis causes ectopic lipid accumulation, we performed in vivo and in vitro experiments in carnitine-deficient JVS mice. We found that carnitine deficiency evoked ectopic lipid accumulation in the kidneys; however, it was insufficient for inducing cell death or injury of PTCs. PTCs exposed to HS+HG in addition to carnitine deficiency underwent cell death with increased production of pro-fibrotic and pro-inflammatory cytokines. Such a condition likely happens in patients with diabetes because dietary sodium intake and total body sodium level are higher, and sodium sensitivity is increased in patients with type 2 diabetes compared with nondiabetic individuals (35, 36).

SDT-f rat is a type 2 diabetes model in which a leptin receptor mutation is introduced into the genetic background of SDT rats (37). Although SDT-f rats develop polyphagia, hyperglycemia, and dyslipidemia from a young age (37), systolic BP likely reaches 130–150 mmHg at most in the SDT-f rats; severe proteinuria and kidney dysfunction are not observed in these animals (38). Salt consumption is increased in patients with diabetes (35), and about 70% of patients with diabetes exhibit hypertension (39), approximately half of which develop DKD (40). Therefore, in order to mimic the human diabetic condition, a high-salt diet was given to uninephrectomized SDT-f rats in the present study. This rat was named SDT-f-DKD, which developed severe hypertension up to 200 mmHg, having massive proteinuria compared with that of SDT-f rats. In addition, SDT-f-DKD rats exhibited severe glomerulosclerosis, formation of nodular lesions in glomerulus, tubular injury, and kidney fibrosis, which recapitulate the typical clinical and histological features of patients with DKD, thus suggesting that SDT-f-DKD rats are a practical rat model of DKD. In this study, although carnitine deficiency or impairment of FAO was not observed in SDT-f rats, SDT-f-DKD rats exhibited carnitine deficiency–induced impairment of FAO and resultant ectopic lipid accumulation in the kidneys.

Furthermore, our time course analysis suggests that OCTN2 expression declines at the early stage of DKD, which temporarily induces upregulation of *Tmlhe* maintaining carnitine homeostasis. However, at the late stage of DKD, reduced *Tmlhe* combined with the decline in OCTN2 causes carnitine deficiency, downregulation of FAO enzymes, and mitochondrial dysfunction. Since salt-sensitive hypertension solely reduced the expression of OCTN2 and *Tmlhe*, it may also contribute to carnitine deficiency in the context of DKD. A high-salt diet has been reported to decrease tissue and plasma concentrations of lysine (41), a precursor of carnitine, which might be another mechanism of the reduction in carnitine biosynthesis (42). Therefore, we propose multifactorial mechanisms for tubular injury in DKD: Salt-sensitive hypertension, hyperglycemia, and hyperlipidemia collectively impair reabsorption and biosynthesis of carnitine, leading to carnitine deficiency. The defects compromise carnitine-induced FAO and mitochondrial respiration, thereby driving the progression of DKD.

Decreased levels of OCTN2, CPT1a, CPT2, and CrAT in SDT-f-DKD rats were drastically restored by l-carnitine supplementation in association with the increased free carnitine level and improvement of carnitine-induced FAO. l-Carnitine supplementation significantly ameliorated tubular injury, kidney dysfunction, and fibrosis with ectopic lipid accumulation in the kidneys of SDT-f-DKD rats. Since KIM-1^+^ injured PTCs exhibited marked reduction in OCTN2 when compared with KIM-1^–^ PTCs, it can be thought that the improvement of tubular injury by l-carnitine supplementation may be associated with preservation of carnitine reabsorption via OCTN2. Furthermore, as FAO impairment progresses, accumulated excessive Acyl-C could inhibit carnitine reabsorption (43), resulting in further carnitine deficiency; thus, the crosstalk between carnitine deficiency and impaired carnitine-induced FAO may form a positive feedback loop. We also found that carnitine deficiency–derived FAO impairment affected mitochondrial biogenesis and induced morphological disruption of mitochondria, both of which were abrogated by l-carnitine supplementation. Given energy status assessed by activation of AMPK was normalized by l-carnitine supplementation, it could improve energy depletion as well via restoring mitochondrial biogenesis and respiratory function.

We observed renoprotective effects of l-carnitine supplementation without any adverse actions in patients with PD; l-carnitine supplementation increased ΔRRF and Δurine volume and reduced ΔLPO. Carnitine-induced FAO impairment may induce a mitochondrial microenvironment that promotes oxidative stress owing to electron “back pressure” and ROS production (44). Moreover, close spatial proximity of mitochondria to membrane structure increased the likelihood of lipid radical formation and peroxidation (45). Considering the observed inverse correlation between serum free carnitine levels and RRF in our PD patients, l-carnitine supplementation may improve carnitine deficiency and carnitine-induced FAO impairment, resulting in reduced ROS production in the patients with PD, including patients with DKD. So far, l-carnitine has been shown to improve several dialysis-related complications, including arrhythmias (46), reduced cardiac output (47), hormone abnormalities (48), and decreased exercise capacity (49). Because of its widely proven safety, l-carnitine supplementation may represent a potentially efficient therapeutic agent for patients with DKD. Further clinical randomized trials will be required in the future.

There are some limitations in our present study. We performed the open-label randomized trial with patients with PD. However, they had already developed end-stage kidney disease with decreased urine volume at the beginning of the trial. It remains unclear whether l-carnitine supplementation may inhibit the development and progression of kidney damage in patients with early-stage DKD. It will be necessary to identify which types of patients are expected to benefit most from l-carnitine supplementation. In addition, l-carnitine overdose has been reported to cause nausea, vomiting, abdominal cramps, and diarrhea. Appropriate dosage could not be examined in the present study; thus, a future study will be needed to address the issue.

**Figure 12:**
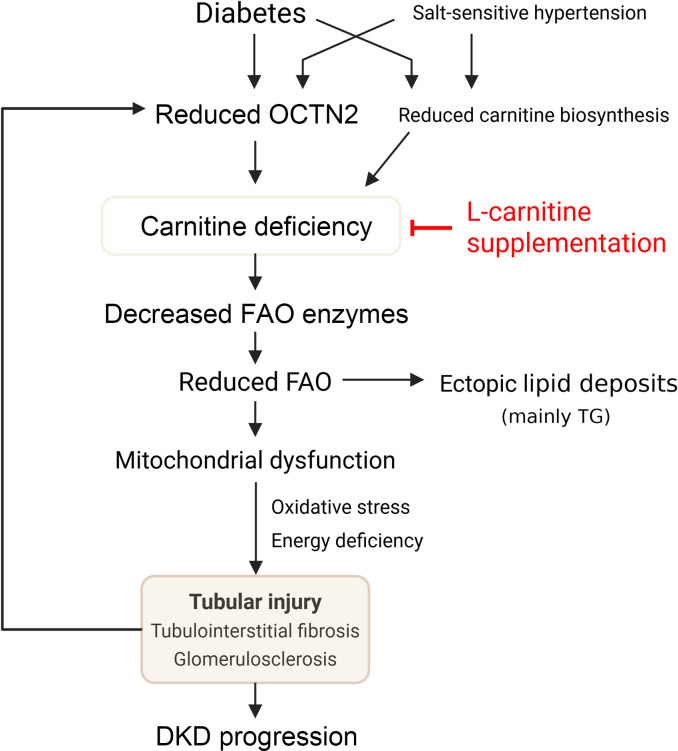
Model of the pathological role of carnitine deficiency in DKD. Diabetes in cooperation with salt-sensitive hypertension downregulates OCTN2 in PTCs, followed by the reduction in carnitine biosynthesis and FAO enzymes, which leads to carnitine-induced FAO impairment. Reduced FAO induces ectopic lipid deposits consisting of mainly triglycerides (TG) and impaired mitochondrial respiration, which promotes tubular injury, kidney fibrosis, and glomerulosclerosis via energy depletion and production of reactive oxygen species (ROS). Tubular injury causes further decline in OCTN2 expression, forming a vicious cycle toward carnitine deficiency. Supplementation with l-carnitine can restore mitochondrial function and reduce lipid accumulation via correcting carnitine concentration, which enables halting of the progression of DKD.

In conclusion, we posit an overall scheme of the role of carnitine in DKD. In concert with salt-sensitive hypertension, hyperglycemia, and hyperlipidemia, decreased OCTN2-mediated reabsorption of carnitine and downregulation of carnitine biosynthesis result in carnitine deficiency, and impairment of carnitine-induced FAO could contribute to tubular injury, kidney dysfunction, and fibrosis with enhanced lipid accumulation in the kidneys of DKD (Figure 12). Supplementation with l-carnitine may be a potent therapeutic strategy for DKD.

## Methods

### Sex as a biological variable.

Our study examined male rodents because they exhibited less variability in phenotype. This approach is consistent with prior studies utilizing a diabetic model and enables clearer mechanistic insights. It is unknown whether the findings are relevant for female rodents. In the studies using human samples, sex was not considered as a biological variable.

### Comparison of ectopic lipid accumulation in patients with MCNS and DKD.

Kidney biopsy specimens, including 10 patients with DKD and 8 age- and sex-matched patients with MCNS who had similar eGFR to the patients with DKD, were obtained from the biopsy database of Department of Nephrology, Kurume University Hospital, from 2017 to 2020. The study protocol was approved by the institutional ethics committees of the Kurume University School of Medicine, Fukuoka, Japan (Ethical No. 22137).

### Investigation of carnitine profile in patients with MCNS and DKD.

A prospective observational study was conducted to investigate carnitine kinetics and profiles in serum and urine of all patients who had been admitted to the Department of Nephrology, Kurume University Hospital, from July 2020 to July 2021. We found 7 patients with MCNS and 38 patients with stage 4 or 5 DKD and then measured carnitine profiles between the 2 groups. The study protocol was approved by the institutional ethics committees of the Kurume University School of Medicine, Fukuoka, Japan (Ethical No. 22137). The detailed profiles and kinetics of carnitine were measured via LC-MS/MS.

### JVS mouse preparation.

JVS mice were provided by Noriyoshi Hashimoto (Division of Transgenic Animal Science, Kanazawa University Advanced Science Research Center, Kanazawa, Japan). JVS mice are used as animal models of systemic carnitine deficiency caused by a mutation in *Slc22a5*. JVS mice develop fatty liver disease, hypoglycemia, and cardiac hypertrophy. Because JVS mice are most likely to die within 21 days after birth owing to severe carnitine deficiency, JVS mice must be injected subcutaneously with 1 mg of l-carnitine–HCl (catalog C0283, Sigma-Aldrich) per day dissolved in saline and neutralized with 0.2 M sodium hydroxide on day 5 through day 28. One group was continuously administered l-carnitine as supplementation until 8 weeks of age, while another group discontinued l-carnitine administration at 4 weeks of age and were injected with carboxymethyl cellulose (catalog C5678, Sigma-Aldrich). Age-matched WT male mice from the same litter (*n* = 5) were used as controls. Plasma, urine, and kidney samples were isolated at 8 weeks of age.

### Isolation of primary PTCs.

PTCs were isolated from WT and JVS mice as previously described (50). Briefly, the kidney cortex was minced and incubated in digestion solution consisting of 1 mg/mL collagenase 4 (catalog 4188, Worthington) and 1 mg/mL trypsin inhibitor (catalog 17075029, Thermo Fisher Scientific) for 20 minutes at 37°C. After adding 10% serum solution to stop the reaction and then removing the supernatant, the pellet was washed with Hank solution (catalog H9394, Sigma-Aldrich). PTC fragments were resuspended in 32% Percoll (catalog P1644, Sigma-Aldrich) using a swing bucket rotor for 10 minutes at 4°C. After washing again with DMEM/F-12, the primary PTCs were seeded and incubated in a 100 cm dish in humidified 95% air/5% CO_2_ at 37°C. The primary PTCs were maintained with DMEM/F-12 supplemented with 10% FBS, penicillin/streptomycin (catalog P4333, Sigma-Aldrich), amphotericin B (catalog 15290018, Thermo Fisher Scientific), insulin-transferrin-sodium selenite solution (catalog I3146, Sigma-Aldrich), hydrocortisone (catalog H0888, Sigma-Aldrich), and vitamin C (catalog Y0000039, MilliporeSigma).

### DKD rodent model.

Male SDT fatty rats and age-matched male SD rats were purchased from CLEA Japan, Inc. SD rats were used as the control group. Uninephrectomy was performed when the rats were 6 weeks old, and 0.3% salt water was administered to create a clinically relevant SDT-f-DKD model. For experiment 1, SD and SDT-f rats were divided into 3 groups as follows: (a) SD rats (*n* = 8), (b) SDT-f rats (*n* = 8), and (c) SDT-f-DKD model rats (*n* = 8). For experiment 2, 3 groups as follows were created: (a) SD rats (*n* = 8), (b) SDT-f-DKD rats treated with vehicle (*n* = 7), and (c) SDT-f-DKD rats treated with l-carnitine supplementation with 0.75% l-carnitine diet (*n* = 6). For time course analysis, SDT-f-DKD rats were sacrificed at 7, 9, 12, and 17 weeks of age. All the rats were placed in metabolic cages for 24 hours for urinalysis during overnight fasting. Systolic BP was measured with a tail cuff sphygmomanometer by using an automated system with a photoelectric sensor (BP-98A; Softron). They were anesthetized with 3% isoflurane (Fujifilm Wako Pure Chemical Corporation) and euthanized with a decapitator after overnight fasting. Serum samples were obtained, and the kidneys were isolated at 17 weeks of age. Plasma, urine, and kidneys were snap-frozen in liquid N_2_ and stored at –80°C. All experimental procedures were conducted in accordance with the National Institutes of Health *Guide for the Care and Use of Laboratory Animals* (National Academies Press, 2011) and approved by the ethical committee of the Kurume University School of Medicine.

### Salt-sensitive hypertension rodent model.

Male 5-week-old Dahl-Iwai S rats were purchased (SLC Inc.) and divided into 3 groups as follows: 0.3% sodium diet as control (Dahl-NS) and 8% sodium diets (Dahl-HS) with or without daily intraperitoneal administration with l-carnitine (6 mg per day) for 6 weeks. Systolic BP was measured with a tail cuff sphygmomanometer by using an automated system with a photoelectric sensor at 5, 8, and 11 weeks of age. Serum, urine, kidney, and liver samples were collected at 11 weeks of age using the same procedures described above.

### Measurement of clinical parameters.

The plasma levels of blood glucose, glycated albumin, BUN, creatinine, total cholesterol, triglycerides, HDL-cholesterol, LDL-cholesterol, and urinary albumin were measured using an auto-analyzer (Nihondenshi Co.). Blood insulin, glucagon, and urinary L-FABP levels were evaluated using a commercially available ELISA kit (Fujifilm Wako Pure Chemical Corporation).

### Western blot analysis.

Whole kidney tissues were homogenized and lysed with 25 mmol/L Tris-HCl (pH 7.4) containing 1% Triton X-100, 0.1% SDS, 2 mmol/L EDTA, and 1% protease inhibitor cocktail (Nacalai Tesque). The supernatant was then separated through SDS-PAGE and transferred to PVDF membranes (Bio-Rad). The membranes were incubated with the following primary antibodies: OCTN2 (Proteintech catalog 16331-1-AP, RRID:AB_2191406), CPT1a (Abcam catalog ab128568, RRID:AB_11141632), CPT2 (Abcam catalog ab181114, RRID:AB_2687503), CrAT (Novus Biologicals catalog NBP1-86616, RRID:AB_11031535), AMPK (Cell Signaling Technology catalog 2532, RRID:AB_330331), p-AMPK (Cell Signaling Technology catalog 2535, RRID:AB_331250), PGC-1α (Proteintech catalog 66369-1-Ig, RRID:AB_2828002), 4HNE (Genox Corporation catalog MHN-020P, RRID:AB_1106814), Complex 1-75kD (MilliporeSimga catalog ABN302, RRID:AB_2915902), and oxidative phosphorylation cocktails (Abcam catalog ab110413, RRID:AB_2629281) and β-actin (Cell Signaling Technology catalog 4967, RRID:AB_330288) overnight. The membranes were then incubated with horseradish peroxidase–conjugated anti-mouse (Cytiva NA931, RRID:AB_772210) or anti-rabbit secondary antibodies (Cell Signaling Technology 7074, RRID:AB_2099233) at 37°C for an hour. Western blots were visualized using Fusion Solo S (Vilber), and protein expression was analyzed using ImageJ (NIH).

### Real-time PCR.

Total RNA was extracted with RNeasy Mini Kit (QIAGEN) from whole kidney or cell lysates, and 1 µg of total RNA was reverse-transcribed using PrimeScript RT Master Mix kit (Takara Bio). Real-time PCR was performed with StepOne (Applied Biosystems). The targeted gene expressions were calculated as a ratio to 18S ribosomal RNA expression. Statistical analysis of the results was performed with the ΔCt (threshold cycle) value (Ct gene of interest – Ct 18S ribosomal RNA). Relative gene expression was obtained from the ΔΔCt method (Ct sample – Ct calibrator). Primer sequences are listed in Supplemental Table 7.

### Histochemical analysis.

Paraffin-embedded, 4 μm sections were stained with PAS, Masson’s trichrome, and Picrosirius red. The stained kidneys were scanned and analyzed using BZ-X800 (Keyence). The percentage of glomerulosclerosis was calculated as the quantity of glomerulosclerosis divided by the total number of glomeruli. The percentage of Oil O Red^+^ area was calculated by dividing Oil Red O^+^ area by kidney cortex. For immunofluorescence staining, 4 μm, paraffin-embedded sections were incubated with TE buffer (pH 9.0), and antigen was retrieved using a pressure cooker. The tissue was blocked with 3% goat serum and 0.1% Triton X-100 in 1% BSA/TBS-Tween and then incubated with the primary antibody for OCTN2 (Proteintech catalog 16331-1-AP, RRID:AB_2191406) and KIM-1 (R&D Systems catalog MAB1817, RRID:AB_2116445) overnight, followed by the incubation with appropriate secondary antibodies, including anti-goat IgG conjugated with Cy5 (Jackson ImmunoResearch catalog 705-175-147, RRID:AB_2340415) and anti-rabbit IgG conjugated with Cy3 (Jackson ImmunoResearch catalog 711-165-152, RRID:AB_2307443). The whole kidney was scanned and the targeted protein^+^ area was analyzed using BZ-X800.

### Histochemical measurements of electron transport chain complex activity.

Fresh kidneys were sectioned at 10 μm thickness and stained for NADH dehydrogenase, SDH, and COX activity. Sections were incubated with 0.8 mg/mL NADH and 1.0 mg/mL nitro blue tetrazolium in phosphate/saline buffer at 37°C for 30 minutes to measure NADH dehydrogenase activity. For SDH activity, sections were incubated with 0.2 M sodium succinate and 1.0 mg/mL nitro blue tetrazolium in phosphate/K-EGTA buffer at 37°C for 30 minutes. For COX activity, sections were incubated with 2.0 mg/mL diaminobenzidine tetrahydrochloride, 2 μg/mL catalase, and 1 mg/mL cytochrome *c* in phosphate buffer adjusted at pH 5.5 at 37°C for 1 hour. The enzymatic reaction was terminated by rinsing with distilled water. Samples for NADH and SDH were mounted with aqueous medium. NADH dehydrogenase, SDH dehydrogenase, and COX activity^+^ area were analyzed, and NADH dehydrogenase, SDH, and COX activity^+^ area/cortex (%) were detected with BZ-X800 analyzer.

### FAO rate measurement.

FAO rate was evaluated with a colorimetric assay kit (Elabscience, E-BC-K784-M) according to the provider’s instruction. Briefly, whole kidney tissues were homogenized with saline, and we collected the supernatant after centrifuge at 10,000*g* for 10 minutes at 4°C. After standardizing the protein concentration (Pierce BCA protein assay kit, Thermo Fisher Scientific), the absorbance at 450 nm was measured. The amount of enzyme in 1 g protein/min that hydrolyzes the substrate to produce NADH at 37°C was defined as 1 unit by the following formula: FAO ability (U/gprot) = (ΔA450-b)/a÷T×1,000/Cpr (ΔA450: OD sample – OD control. T: reaction time, 30 minutes. Cpr: concentration of protein in sample).

### Measurement of carnitine profiles in the kidneys.

Kidney tissues were homogenized and dissolved with 1 M potassium dihydrogen phosphate, and carnitine internal standard solution (Cambridge Isotope Laboratories, Inc. NSK-B) was dissolved in acetonitrile/methanol. After a centrifugation at 14,000*g* for 10 minutes, acetonitrile/methanol was added to the supernatant. Following centrifugation at 14,000*g* for 20 minutes, the supernatants were used. HPLC separation was performed by hydrophilic interaction chromatography with column (SeQuant, ZIC-HILIC 2.1 × 150 mm, particle size 3.5 μm). Then, 10 mM formic acid and acetonitrile were added to the eluted solvents. A quadrupole mass spectrometer (API 3000, Applied Biosystems) was used for MS/MS (selected reaction monitoring method). The ion source was a turbo spray in positive mode at 450°C with 7,000 mL/min of turbo gas flow rate.

### EM.

Kidney sections were fixed with 2% paraformaldehyde and 2.5% glutaraldehyde in 0.1 M phosphate-buffered saline buffer (PBS; pH 7.4) at 4°C for 7 minutes overnight. Subsequently, they were incubated with 2% osmium tetroxide in PBS in ice for 2 hours. The fixed samples were dehydrated, infiltrated, embedded in Quetol-812 (Nisshin EM), and polymerized at 60°C for 48 hours. Resin blocks were semithin-sectioned into 1.5 μm sections with a glass knife by using an ultramicrotome (Leica) and stained with 0.5% toluidine blue. The blocks were then ultrathin-sectioned into 70 nm sections with a diamond knife by using an ultramicrotome. The sections were placed on copper grids, stained with 2% uranyl acetate at room temperature for 15 minutes, rinsed with distilled water, and stained with lead stain solution (Sigma-Aldrich) at room temperature for 3 minutes. The grids were observed using a JEOL JEM-1200EX transmission electron microscope at an acceleration voltage of 80 kV.

### Study design for a single-center clinical trial in patients undergoing PD.

A prospective, open-label, randomized clinical trial was conducted at Kurume University Hospital, Fukuoka, Japan. Patients with PD were randomly assigned into control and l-carnitine groups. We administered l-carnitine 750 mg 3 times a day orally for 6 months. Informed consent was obtained from all enrolled patients, and the study protocol was approved by the Institutional Ethics Committee of Kurume University School of Medicine, Japan (Ethical No. 16047). This trial was registered in the University Hospital Medical Information Network clinical trial database (UMIN000031514). Exclusion criteria in this study were as follows: (a) less than 20 years of age, (b) history of hemodialysis (HD), (c) undergoing hybrid therapy of PD and HD, and (d) less than 3 months of PD history. All samples, including serum, urine, and PD dialysate, were collected when admitted for the evaluation of standard peritoneal equilibration test, and PD Adequest Weekly Kt/V and RRF were calculated by PD Adequest 2.0, a specific software (Baxter Healthcare). Carnitine profiles in serum and urine were measured by LC-MS/MS. Prescription of PD dialysate was changed only when PD efficiency was insufficient in accordance with International Society of Peritoneal Dialysis guidelines (51).

### Statistics.

Data were presented as the mean ± SEM or SD. The presence or absence of diabetes mellitus and use of renin-angiotensin system inhibitors were coded as dummy variables. Univariate regression analysis was conducted to evaluate correlations. Unpaired, 2-tailed Student’s *t* test was performed when comparing 2 groups. One-way ANOVA followed by post hoc Turkey’s test was performed to assess the significant differences when comparing over 3 groups. Data were statistically analyzed using GraphPad Prism version 8.0 or JMP Pro version 14 software (SAS Institute Inc.). Statistical significance was set at *P* < 0.05.

### Study approval.

All animal experiments were approved by the Institutional Animal Care and Use Committee of Kurume University School of Medicine. Human samples were obtained under protocols approved by the institutional ethics committees of the Kurume University School of Medicine, Fukuoka, Japan (Ethical No. 22137 and 16047). All necessary patient/participant written informed consent was obtained.

### Data availability.

All data supporting the findings of this study are provided in the accompanying Supporting Data Values Excel file. Additional information or clarification is available from the corresponding author upon reasonable request.

## Author contributions

SI and KF conceived the original idea of this study. SI, K Taguchi, GK, SK, TM, Y Yamashita, Y Yokota, YN, YK, K Tashiro, and KO performed the experiments. K Taguchi, MS, SY, and KF supervised the experiments. K Taguchi wrote most of the initial draft of the manuscript, and SY and KF edited the manuscript. All the authors have read and approved the final version of the manuscript.

## Supplementary Material

Supplemental data

Unedited blot and gel images

Supporting data values

## Figures and Tables

**Table 1:**
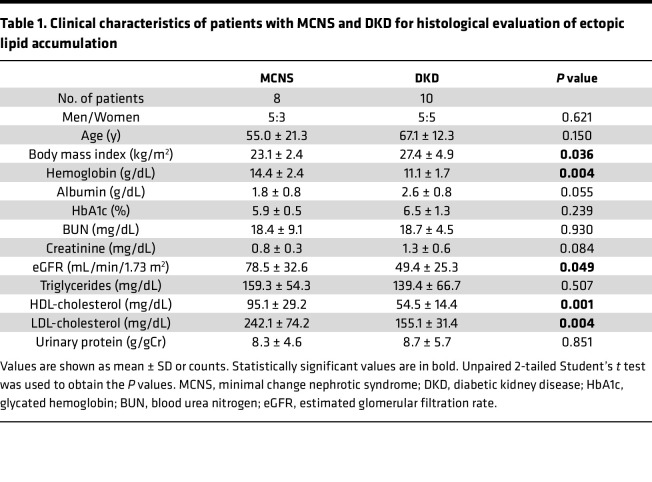
Clinical characteristics of patients with MCNS and DKD for histological evaluation of ectopic lipid accumulation

**Table 2:**
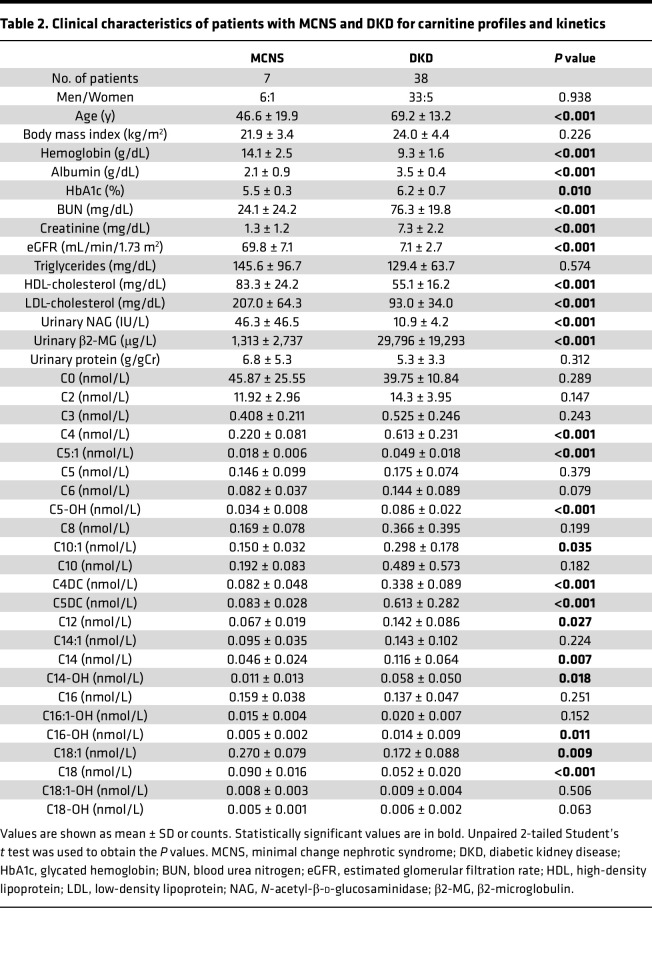
Clinical characteristics of patients with MCNS and DKD for carnitine profiles and kinetics

## Version 1 05/22/2025

In-Press Preview

## Version 2 07/08/2025

Electronic publication
