# Supplementary material for: Involvement of impaired carnitine-induced fatty acid oxidation in experimental and human diabetic kidney disease
Source: JCI Insight. 2025 May 22;10(13):e179362. doi: 10.1172/jci.insight.179362 (PMC12288908; doi:10.1172/jci.insight.179362)
Supplement: Supplemental data [file jciinsight-10-179362-s040.pdf]

Supplementary Figure 1

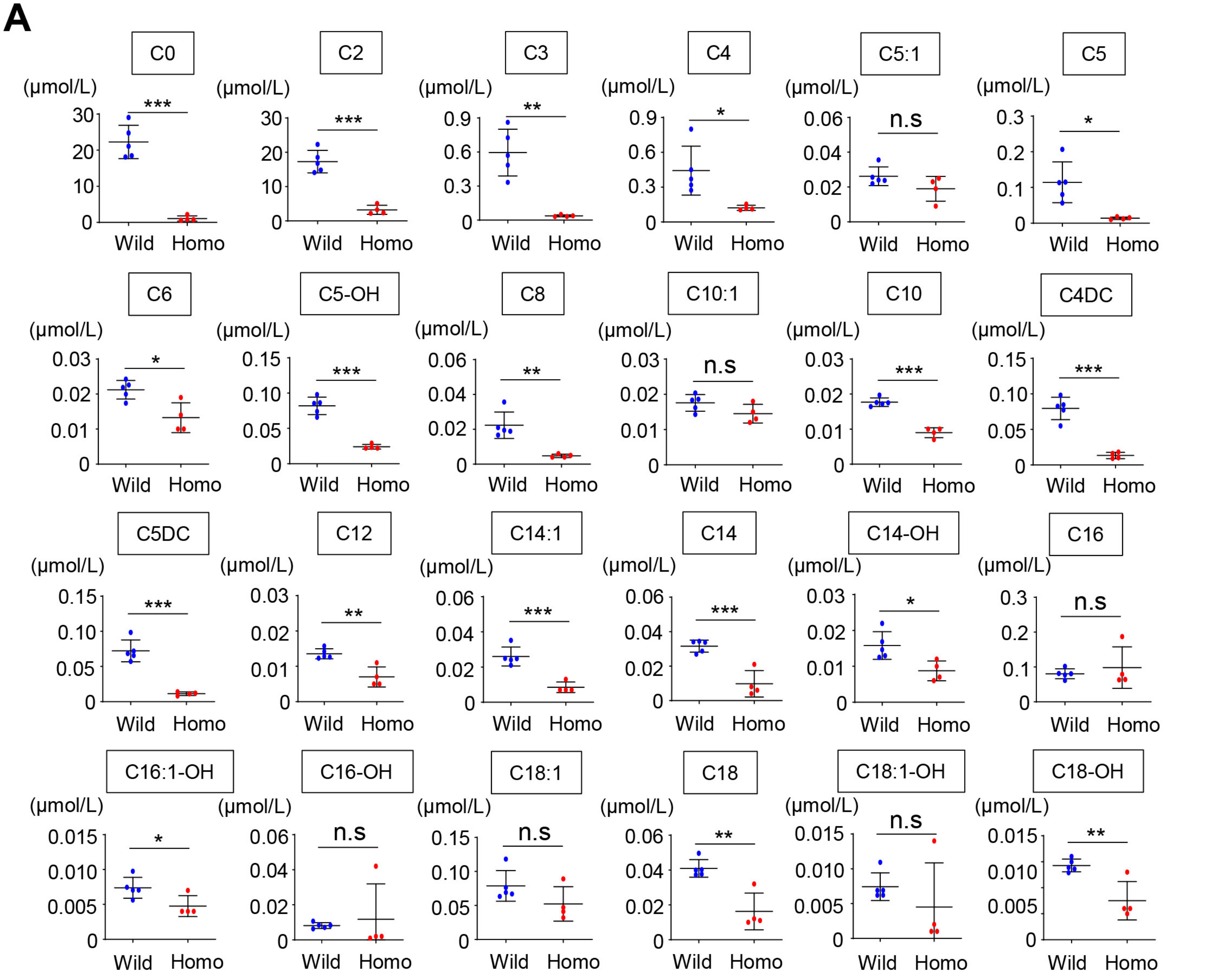

Supplementary Figure 1. Detailed carnitine profiling in JVS mice
(A) LC-MS/MS provided carnitine profiles with plasma in JVS mice (n=4) and wild-type mice (n=5). Data are presented as means ± SD. *P < 0.05, **P < 0.01, ***P < 0.001. JVS, juvenile visceral steatosis; LC-MS/MS, liquid chromatograph-mass spectrometry.

Supplementary Figure 2

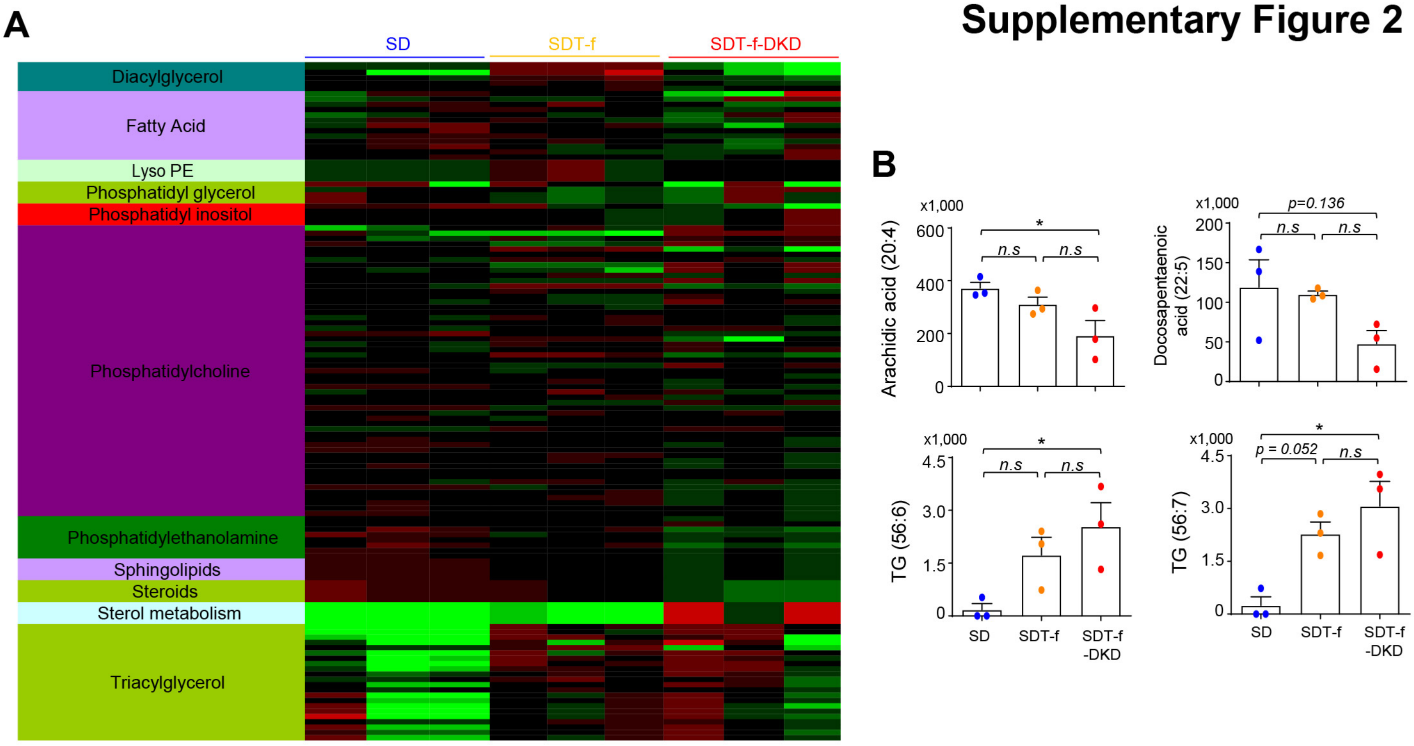

Supplementary Figure 2. Ectopic lipid accumulates in the kidney of DKD rats.
(A) Heat map analysis of lipid metabolites based on fold change in SD (n=3), SDT-f (n=3), and SDT-f-DKD (n=3). (B) Corresponding data of arachidic acid (20:4), docosapentaenoic acid (22:5), TG (56:6), and TG (57;6). Data are presented as means ± SD. *P < 0.05. DKD, diabetic kidney disease; SD, Sprague-Dawley; SDT, spontaneously diabetic torii, SDT-f, spontaneously diabetic torii-fatty; TG, triglycerides.

Supplementary Figure 3

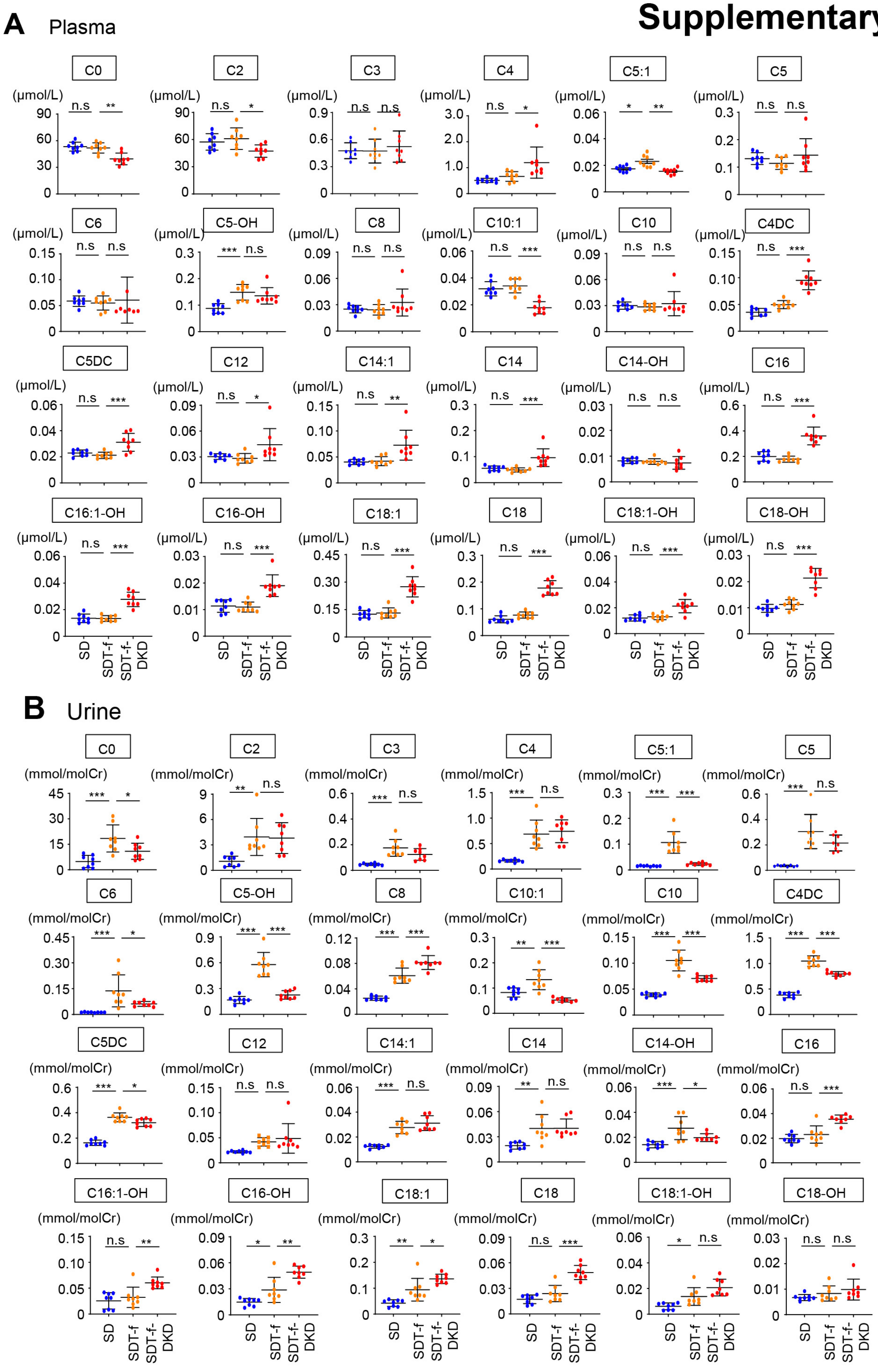

Supplementary Figure 3. Detailed carnitine profiling in SD, SDT-f, and SDT-f-DKD rats.
(A) LC-MS/MS provided the carnitine profiles of the plasma of SD (n=8), SDT-f (n=8), and SDT-f-DKD rats (n=8). (B) Urine carnitine profiles of the urine of SD (n=8), SDT-f (n=8), and SDT-f-DKD rats (n=8). Data are presented as means ± SD. *P < 0.05, **P < 0.01, ***P < 0.001. SD, Sprague-Dawley; SDT, spontaneously diabetic torii, SDT-f, spontaneously diabetic torii-fatty; DKD, diabetic kidney disease; LC-MS/MS, liquid chromatograph-mass spectrometry.

A Plasma

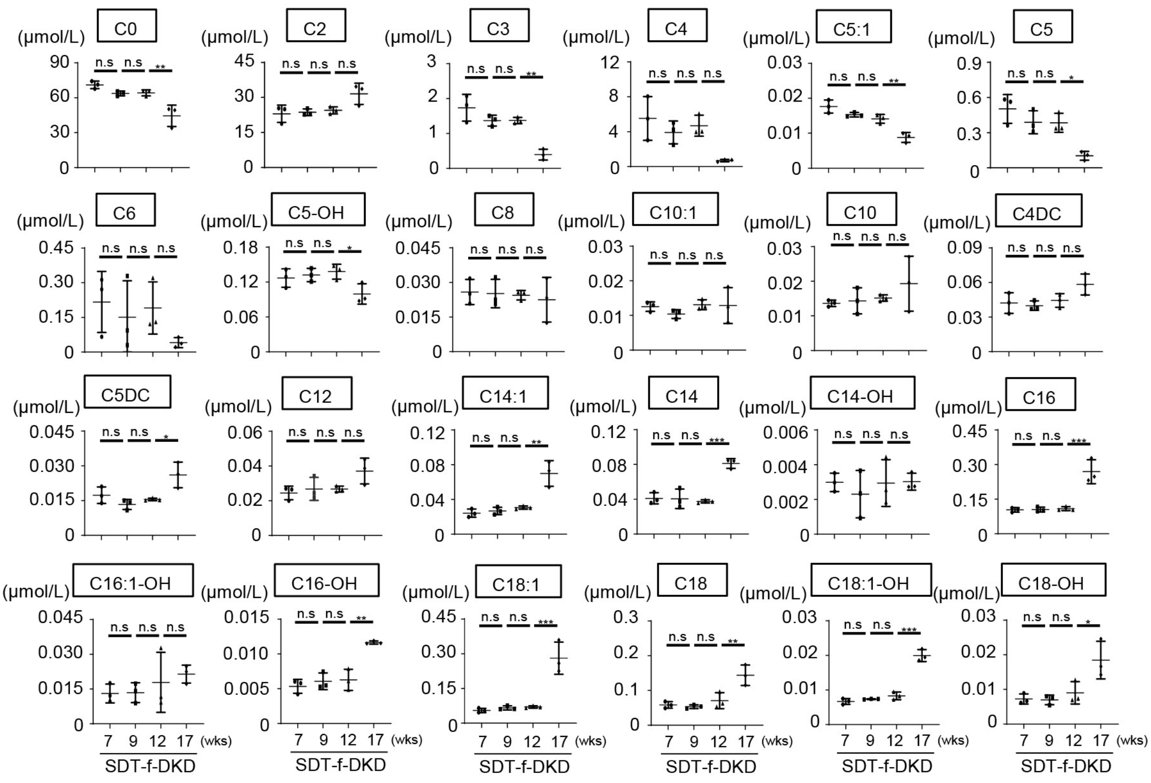

B Urine

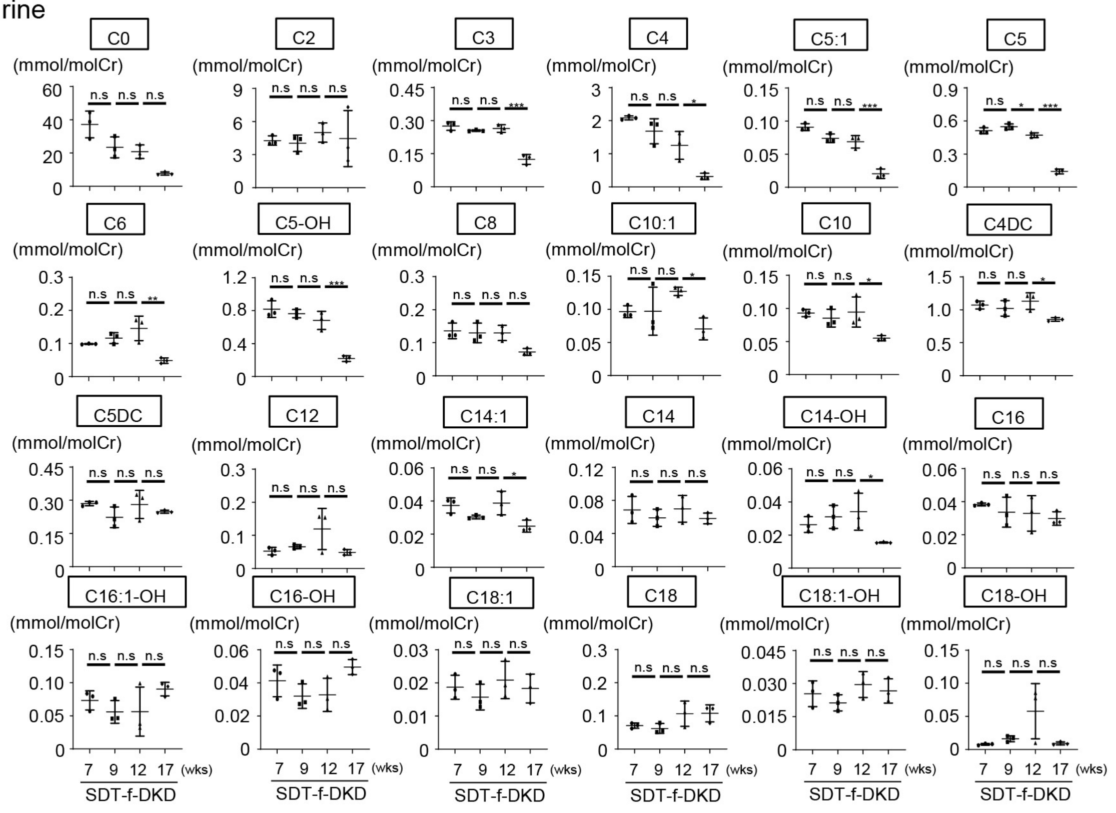

Supplementary Figure 4. Detailed carnitine profiling in over the time-course of SDT-f-DKD rats
(A) LC-MS/MS provided the carnitine profiles of the plasma and (B) urine in SDT-f-DKD rats at 7, 9, 12 and 17 weeks of age. n=3, respectively. Data are presented as means ± SD. *P < 0.05, **P < 0.01, ***P < 0.001. SDT-f, spontaneously diabetic torii-fatty; DKD, diabetic kidney disease; L-Car, L-carnitine; LC-MS/MS, liquid chromatograph-mass spectrometry.

Supplementary Figure 5

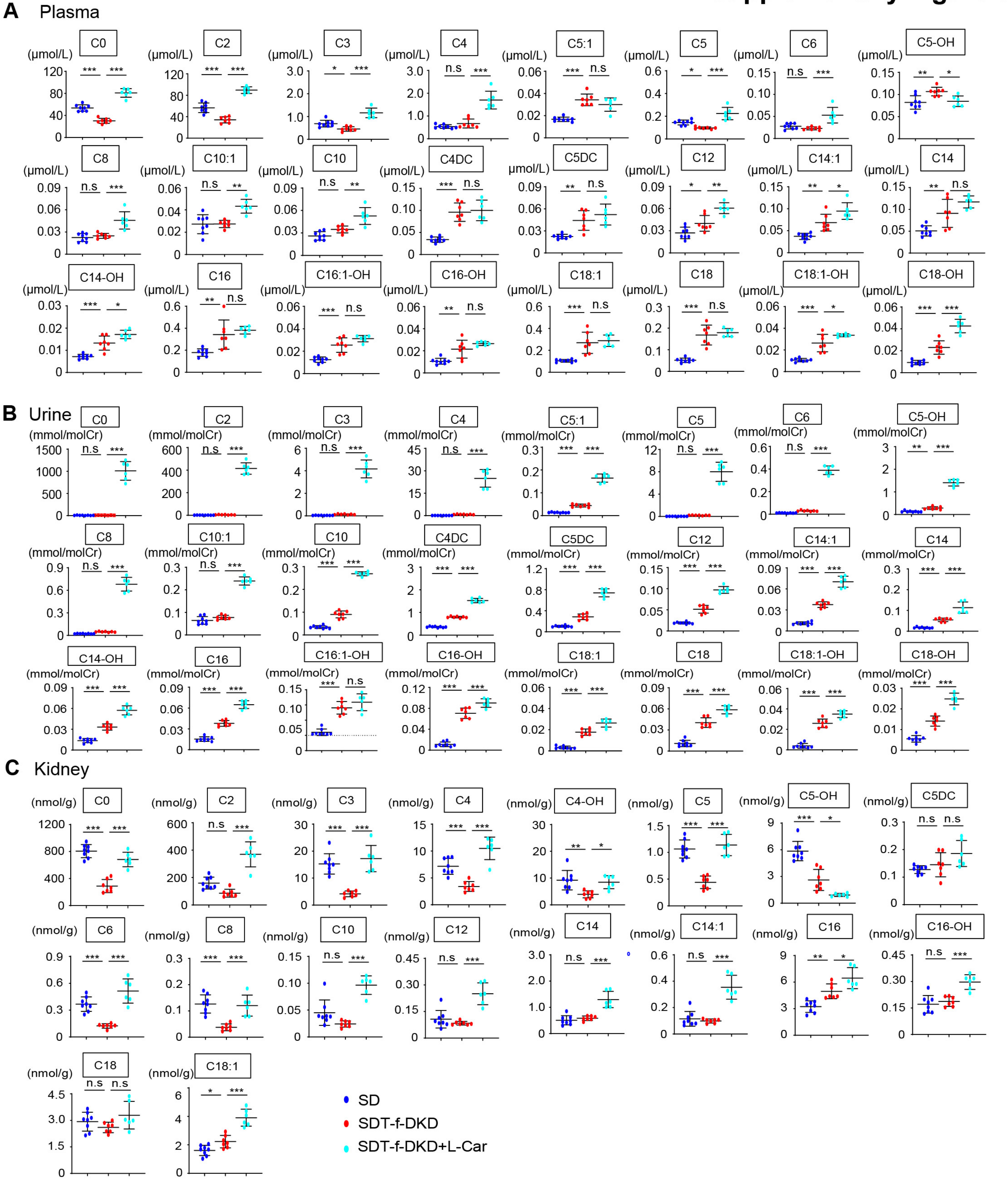

Supplementary Figure 5. Detailed carnitine profiling of SDT-f-DKD rats treated with L-carnitine supplementation. (A) LC-MS/MS provided the carnitine profiles of the plasma of SD (n=8), SDT-f-DKD (n=7), and SDT-f-DKD+L-Car rats (n=6). (B) Urine carnitine profiles of the urine of SD (n=8), SDT-f-DKD (n=7), and SDT-f-DKD rats+L-Car rats (n=6). (C) Carnitine profiling of the kidney cortex of SD (n=8), SDT-f-DKD (n=7), and SDT-f-DKD+L-Car rats (n=6). Data are presented as means ± SD. *P < 0.05, **P < 0.01, ***P < 0.001. SDT, spontaneously diabetic torii, SDT-f, spontaneously diabetic torii-fatty; DKD, diabetic kidney disease; SD, Sprague-Dawley; LC-MS/MS, liquid chromatograph-mass spectrometry; L-Car, L-carnitine.

Supplementary Figure 6

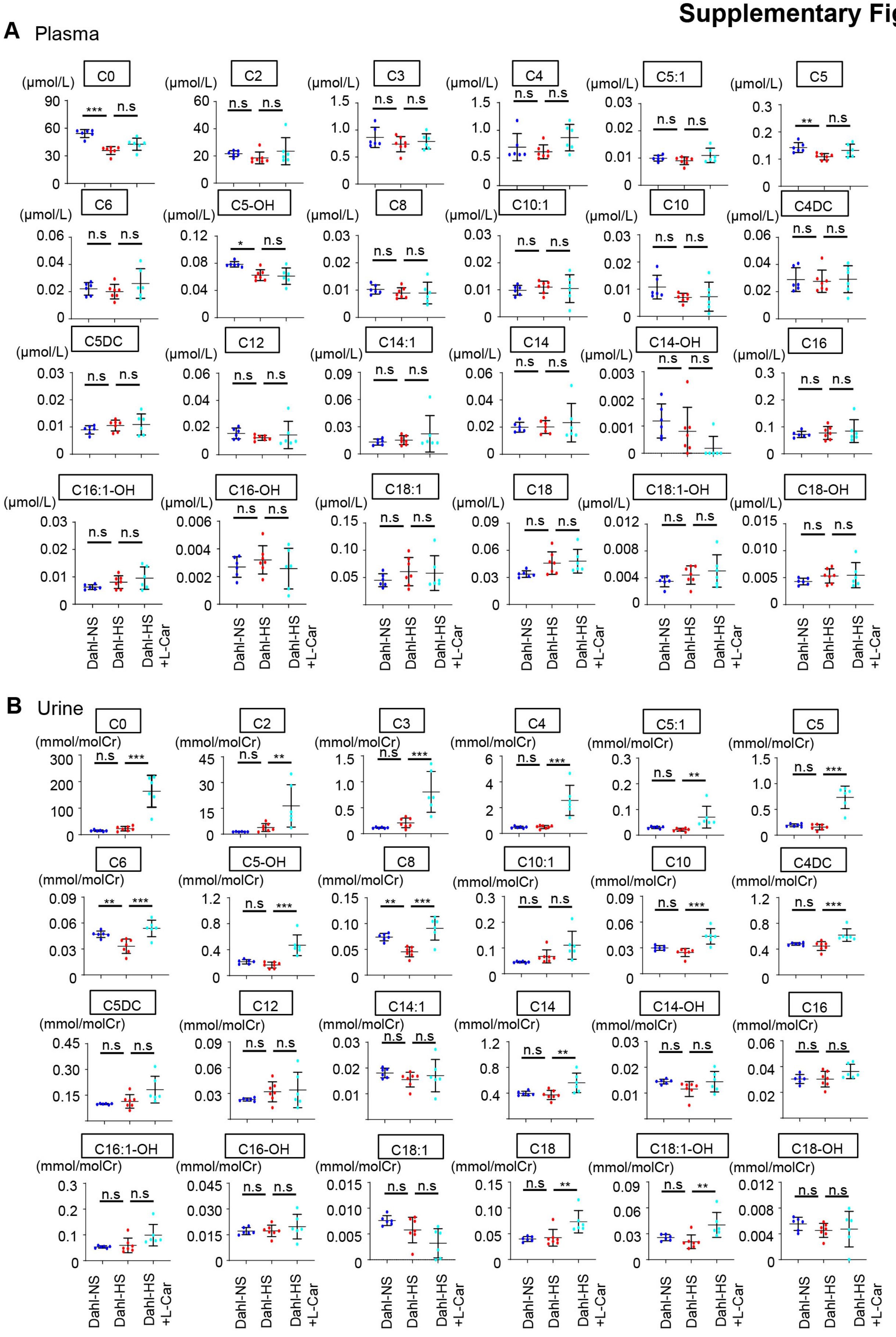

Supplementary Figure 6. Detailed carnitine profiling in Dahl rat treated with or without L-Car.
(A) LC-MS/MS provided the carnitine profiles of the plasma of Dahl-NS (n=6), Dahl-HS (n=7), and Dahl-HS+L-car (n=6). (B) Urine carnitine profiles of the urine of Dahl-NS (n=6), Dahl-HS (n=7), and Dahl-HS+L-car (n=6). Data are presented as means ± SD. *P < 0.05, **P < 0.01, ***P < 0.001. NS, normal salt; HS, high salt; Car, carnitine; LC-MS/MS, liquid chromatograph-mass spectrometry.

Supplementary Table 1. Characteristics of SDT-fatty rats and SDT-fatty DKD rats

	SD	SDT-f	SDT-f-DKD
Number	8	8	8
Body weight (g)	516.3±43.8	539.4±38.5	632.4±57.2 $^{\#\#}$
Glycated albumin (%)	7.9±0.5	24.2±3.9***	6.9±3.6$^{\#\#\#}$
BUN (mg/dL)	14.4±1.8	26.1±8.8**	35.8±5.7$^{\#}$
Creatinine (mg/dL)	0.29±0.04	0.22±0.05	0.54±0.08$^{\#\#\#}$
Total cholesterol (mg/dL)	51.0±6.1	131.4±11.5***	278.9±62.2$^{\#\#\#}$
Triglycerides (mg/dL)	47.8±18.6	297.6±37.1*	771.4±284.8 $^{\#\#\#}$
HDL-cholesterol (mg/dL)	23.3±2.0	56.3±3.3***	55.5±6.6
LDL-cholesterol (mg/dL)	7.4±1.8	9.1±3.0	28.0±20.5$^{\#}$
Insulin (pg/mL)	219.5±209.0	2075.7±1091.7***	2093.0±484.2
Glucagon (pmol/L)	3.5±2.5	2.4±1.4	10.5±5.6$^{**\ \#\#\#}$
Urinary albumin (mg/gCr)	0.01±0.00	3.52±0.49***	5.67±2.34 $^{\#}$

Data are shown as mean ± SD.

Abbreviations: SD=Sprague-Dawley, SDT-f=Spontaneously Diabetic Torii-fatty, DKD=Diabetic Kidney Disease, HDL=high-density lipoprotein, LDL=low-density lipoprotein.

* $p < 0.05$, ** $p < 0.01$, *** $p < 0.001$ vs SD, $^{\#}$ $p < 0.05$, $^{\#\#}p<0.01$, $^{\#\#\#}p<0.001$ vs SDT-f

Supplementary Table 2. Clinical characteristics over time-course of SDT-f-DKD rats

SDT-f-DKD rats	7W	9W	12W	17W
Number	3	3	3	3
Body weight (g)	320.5 ± 4.1	443.6 ± 5.6**	582.4 ± 35.5***##	736.8 ± 43.3***###††
BUN (mg/dL)	38.3 ± 5.0	30.0 ± 1.0	30.7 ± 4.2	29.7 ± 8.7
Creatinine (mg/dL)	0.29 ± 0.02	0.29 ± 0.02	0.30 ± 0.03	0.44 ± 0.05**##††
Total cholesterol (mg/dL)	119.7 ± 4.9	109.0 ± 13.0	161.3 ± 18.6	265.3 ± 99.8*#
Triglycerides (mg/dL)	295.3 ± 29.7	325.3 ± 28.2	437.7 ± 92.3	661.7 ± 446.1
HDL-cholesterol (mg/dL)	85.3 ± 6.7	84.7 ± 9.8	114.7 ± 9.5*#	131.0 ± 9.5**##
LDL-cholesterol (mg/dL)	16.7 ± 0.6	9.7 ± 2.1	19.3 ± 4.0	48.7 ± 32.2
Urinary albumin (mg/gCr)	0.65 ± 0.32	2.11 ± 1.60	5.34 ± 2.35*	7.19 ± 0.46**#

Data are shown as mean ± SD.

Abbreviations: SDT-f=Spontaneously Diabetic Torii-fatty, DKD=Diabetic Kidney Disease, HDL=high-density lipoprotein, LDL=low-density lipoprotein.

* $p < 0.05$, ** $p < 0.01$, *** $p < 0.001$ vs 7W, # $p < 0.05$, ## $p<0.01$, ### $p<0.001$ vs 9W, ††: $P<0.01$ vs 12W

Supplementary Table 3. Characteristics of SDT-fatty DKD rats with or without L-Carnitine treatment

	SD	SDT-f-DKD	SDT-f-DKD + L-Carnitine
Number	8	7	6
Body weight (g)	547.3 ± 29.9	676.6 ± 51.3***	628.5 ± 67.1
Glycated albumin (%)	7.7 ± 0.9	6.7 ± 2.9	8.5 ± 2.1
BUN (mg/dL)	15.4 ± 2.6	38.7 ± 9.5***	30.0 ± 5.8
Creatinine (mg/dL)	0.30 ± 0.05	0.67 ± 0.20***	0.47 ± 0.11#
Total cholesterol (mg/dL)	47.8 ± 8.8	337.4 ± 74.4***	295.2 ± 83.5
Triglycerides (mg/dL)	49.4 ± 20.7	643.6 ± 199.8***	564.3 ± 161.3
HDL-cholesterol (mg/dL)	23.3 ± 3.1	68.6 ± 5.5***	66.3 ± 10.6
LDL-cholesterol (mg/dL)	4.6 ± 1.2	33.7 ± 18.8**	24.7 ± 18.5
Insulin (pg/mL)	123.7 ± 96.7	2041.1 ± 692.7***	1846.4 ± 841.3
Glucagon (pmol/L)	3.6 ± 1.0	17.5 ± 5.4***	11.4 ± 3.6#
Urinary albumin (mg/gCr)	0.01 ± 0.00	8.99 ± 2.04***	6.15 ± 1.51#

Data are shown as mean ± SD.

Abbreviations: SD=Sprague-Dawley, SDT-f=Spontaneously Diabetic Torii-fatty, DKD=Diabetic Kidney Disease, HDL=high-density lipoprotein, LDL=low-density lipoprotein.

* $p < 0.05$, ** $p < 0.01$, *** $p < 0.001$ vs SD, # $p < 0.05$ vs SDT-f-DKD

Supplementary Table 4. Characteristics of Dahl-S rats with or without L-Carnitine treatment

	Dahl-NS	Dahl-HS	Dahl-HS + L-Carnitine
Number	6	7	6
Body weight (g)	323.6 ± 11.4	283.9 ± 34.4	285.1 ± 44.2
BUN (mg/dL)	17.2 ± 1.7	27.1 ± 8.7	27.5 ± 10.2
Creatinine (mg/dL)	0.26 ± 0.02	0.35 ± 0.10	0.30 ± 0.06
Total cholesterol (mg/dL)	58.1 ± 1.8	104.3 ± 11.1***	99.2 ± 15.8***
Triglycerides (mg/dL)	173.5 ± 11.6	213.9 ± 118.5***	218.8 ± 108.2
HDL-cholesterol (mg/dL)	24.2 ± 0.7	36.6 ± 9.6*	48.7 ± 6.7*** #
LDL-cholesterol (mg/dL)	11.2 ± 1.2	22.3 ± 5.3**	27.3 ± 8.2***
Urinary albumin (mg/gCr)	0.77 ± 0.56	3.54 ± 1.62	6.86 ± 4.77**

Data are shown as mean ± SD.

Abbreviations: Dahl-S=Dahl salt-sensitive, NS=Normal Salt, HS=High Salt, HDL=high-density lipoprotein, LDL=low-density lipoprotein.

* $p < 0.05$, ** $p < 0.01$, *** $p < 0.001$ vs Dahl-S-NS, # $p < 0.05$, ### $p<0.001$ vs Dahl-S-HS

Supplementary Table 5. Effects of L-Carnitine Supplementation on Clinical Variables in Patients with Peritoneal Dialysis

	Control group (n=12)	L-carnitine group (n=12)	p-value between groups
Men : Women	7: 5	11 : 1	0.063
Age (years old)	63.1 ± 9.8	62.3 ± 10.5	0.858
Body Mass Index (kg/m^2)	22.6 ± 2.5	24.8 ± 2.6	**0.044**
PD duration (day)	635.1 ± 440.2	445.6 ± 348.4	0.255
Diabetes (No.) (-/+)	(11/1)	(6/6)	**0.024**
ACE-I or ARB (No.) (-/+)	(3/9)	(3/9)	1.000
Dialysis efficiency parameters			
PD efficiency (weekly-Kt/V)			
Baseline	1.03 ± 0.37	1.08 ± 0.21	0.646
Post-treatment	0.96 ± 0.26	1.17 ± 0.28	0.486
p value vs. baseline	0.588	0.408	
ΔPD efficiency	-0.07 ± 0.19	0.09 ± 0.20	0.066
RRF (weekly-Kt/V)			
Baseline	0.86 ± 0.51	0.77 ± 0.34	0.607
Post-treatment	0.59 ± 0.46	0.74 ± 0.37	0.386
p value vs. baseline	0.194	0.872	
ΔRRF	-0.26 ± 0.32	-0.02 ± 0.22	**0.043**
Dialysate volume (L/day)			
Baseline	4.67 ± 1.12	5.73 ± 1.18	**0.034**
Post-treatment	4.74 ± 1.11	5.88 ± 1.21	**0.026**
p value vs. baseline	0.870	0.762	
ΔDialysate volume	0.08 ± 0.70	0.15 ± 0.49	0.763
Urine volume (L/day)			
Baseline	1.31 ± 0.66	1.18 ± 0.83	0.680
Post-treatment	0.94 ± 0.57	1.28 ± 0.76	0.232
p value vs. baseline	0.159	0.763	
ΔUrine volume	-0.37 ± 0.47	0.10 ± 0.32	**0.010**
D/P Creatinine			
Baseline	0.60 ± 0.11	0.61 ± 0.11	0.912
Post-treatment	0.64 ± 0.15	0.63 ± 0.10	0.863
p value vs. baseline	0.522	0.655	
ΔD/P Creatinine	0.03 ± 0.09	-0.03 ± 0.23	0.363
D/D0 Glucose			
Baseline	0.42 ± 0.10	0.42 ± 0.08	0.964
Post-treatment	0.39 ± 0.11	0.40 ± 0.09	0.762

p value vs. baseline	0.557	0.772	
ΔD/D0 Glucose	-0.03 ± 0.06	-0.04 ± 0.08	0.496
Biomedical parameters			
Hemoglobin (g/dL)			
Baseline	11.3 ± 1.4	11.8 ± 1.8	0.530
Post-treatment	11.1 ± 1.1	11.2 ± 1.6	0.906
p value vs. baseline	0.664	0.416	
ΔHemoglobin	-0.23 ± 1.48	-0.58 ± 1.16	0.517
Albumin (g/dL)			
Baseline	3.3 ± 0.4	3.3 ± 0.2	0.910
Post-treatment	3.2 ± 0.5	3.3 ± 0.2	0.618
p value vs. baseline	0.530	0.766	
ΔAlbumin	-0.12 ± 0.27	-0.03 ± 0.25	0.397
BUN (mg/dL)			
Baseline	54.6 ± 14.3	50.5 ± 9.5	0.412
Post-treatment	65.7 ± 7.0	58.4 ± 10.5	0.057
p value vs. baseline	**0.025**	0.066	
ΔBUN	11.1 ± 13.8	7.9 ± 8.0	0.499
Creatinine (mg/dL)			
Baseline	9.7 ± 4.5	8.9 ± 2.3	0.578
Post-treatment	11.6 ± 5.0	9.5 ± 2.6	0.201
p value vs. baseline	0.348	0.601	
ΔCreatinine	1.9 ± 2.0	0.5 ± 0.8	**0.044**
Uric acid (mg/dL)			
Baseline	6.2 ± 1.1	6.4 ± 1.1	0.730
Post-treatment	7.0 ± 1.6	6.6 ± 1.1	0.518
p value vs. baseline	0.193	0.626	
ΔUric acid	0.8 ± 0.9	0.2 ± 0.4	0.065
LDL-cholesterol (mg/dL)			
Baseline	102.6 ± 24.1	86.4 ± 23.6	0.111
Post-treatment	106.8 ± 28.8	91.7 ± 22.5	0.165
p value vs. baseline	0.699	0.580	
ΔLDL-cholesterol	4.3 ± 21.1	5.3 ± 9.0	0.878
Triglycerides (mg/dL)			
Baseline	133.6 ± 40.8	154.3 ± 76.2	0.416
Post-treatment	131.0 ± 41.7	159.5 ± 71.1	0.244
p value vs. baseline	0.880	0.863	
ΔTriglycerides	-2.6 ± 24.3	5.3 ± 45.1	0.612
LPO (nmol/mL)			
Baseline	4.2 ± 0.6	4.6 ± 0.8	0.139

Post-treatment	4.5 ± 0.9	4.0 ± 0.6	0.175
p value vs. baseline	0.314	0.068	
ΔLPO	0.32 ± 0.81	-0.58 ± 0.67	**0.007**
Urine L-FABP (ng/mL)			
Baseline	78.7 ± 29.3	118.4 ± 69.3	0.081
Post-treatment	98.2 ± 65.0	94.3 ± 39.6	0.862
p value vs. baseline	0.355	0.307	
ΔUrine L-FABP	19.5 ± 53.7	-24.1 ± 65.0	0.087
Urine osmolality (mOsm/L)			
Baseline	256.7 ± 52.3	277.4 ± 52.4	0.342
Post-treatment	259.8 ± 53.1	274.8 ± 84.1	0.604
p value vs. baseline	0.887	0.929	
ΔUrine osmolality	3.1 ± 37.0	-0.26 ± 104.0	0.860

Values are shown as mean ± SD or range.

PD=Peritoneal Dialysis, ACE-I=angiotensin converting enzyme inhibitor, ARB=angiotensin II receptor blocker, RRF=residual renal function, D/P Creatinine=dialysate to plasma creatinine ratio, D/D0 Glucose=4 to 0-hours dialysate glucose ratio, BUN=blood urea nitrogen, LDL=low-density lipoprotein, LPO=lipid peroxides, L-FABP=liver-type fatty acid binding protein.

Supplementary Table 6. Carnitine profiles in Patients with Peritoneal Dialysis with or without L-carnitine treatment

	Control group (n=12)	L-carnitine group (n=12)	p-value between groups
C0 (nmol/L)			
Baseline	27.569 ± 7.111	28.318 ± 7.240	0.801
Post-treatment	27.368 ± 6.140	116.451 ± 28.497	**<0.001**
p value vs. baseline	0.941	**<0.001**	
ΔC0	-0.202 ± 4.573	88.133 ± 27.874	**<0.001**
C2 (nmol/L)			
Baseline	9.057 ± 1.692	9.099 ± 2.153	0.958
Post-treatment	8.261 ± 1.235	34.909 ± 13.394	**<0.001**
p value vs. baseline	0.202	**<0.001**	
ΔC2	-0.796 ± 1.284	25.810 ± 12.816	**<0.001**
C3 (nmol/L)			
Baseline	0.289 ± 0.117	0.283 ± 0.103	0.884
Post-treatment	0.244 ± 0.070	1.580 ± 0.672	**<0.001**
p value vs. baseline	0.265	**<0.001**	
ΔC3	-0.045 ± 0.093	1.297 ± 0.628	**<0.001**
C4 (nmol/L)			
Baseline	0.476 ± 0.189	0.476 ± 0.130	0.996
Post-treatment	0.525 ± 0.171	1.759 ± 0.384	**<0.001**
p value vs. baseline	0.516	**<0.001**	
ΔC4	0.049 ± 0.159	1.282 ± 0.367	**<0.001**
C5:1 (nmol/L)			
Baseline	0.061 ± 0.016	0.057 ± 0.010	0.521
Post-treatment	0.066 ± 0.013	0.078 ± 0.013	**0.024**
p value vs. baseline	0.407	**<0.001**	
ΔC5:1	0.005 ± 0.012	0.021 ± 0.011	**0.002**
C5 (nmol/L)			
Baseline	0.141 ± 0.035	1.143 ± 3.463	0.327
Post-treatment	0.136 ± 0.032	0.380 ± 0.127	**<0.001**
p value vs. baseline	0.720	0.454	
ΔC5	-0.005 ± 0.037	-0.763 ± 3.354	0.442
C6 (nmol/L)			
Baseline	0.140 ± 0.069	0.100 ± 0.028	0.077
Post-treatment	0.103 ± 0.032	0.505 ± 0.274	**<0.001**
p value vs. baseline	0.103	**<0.001**	
ΔC6	-0.037 ± 0.079	0.405 ± 0.261	**<0.001**
C5-OH (nmol/L)			
Baseline	0.088 ± 0.029	0.079 ± 0.014	0.347
Post-treatment	0.097 ± 0.022	0.146 ± 0.037	**<0.001**

p value vs. baseline	0.405	**<0.001**	
ΔC5-OH	0.009 ± 0.016	0.067 ± 0.029	**<0.001**
C8 (nmol/L)			
Baseline	0.090 ± 0.027	0.125 ± 0.045	**0.031**
Post-treatment	0.092 ± 0.030	0.330 ± 0.090	**<0.001**
p value vs. baseline	0.879	**<0.001**	
ΔC8	0.002 ± 0.031	0.205 ± 0.078	**<0.001**
C10:1 (nmol/L)			
Baseline	0.107 ± 0.032	0.162 ± 0.059	**0.010**
Post-treatment	0.114 ± 0.037	0.334 ± 0.085	**<0.001**
p value vs. baseline	0.608	**<0.001**	
ΔC10:1	0.007 ± 0.033	0.173 ± 0.086	**<0.001**
C10 (nmol/L)			
Baseline	0.097 ± 0.038	0.162 ± 0.068	**0.008**
Post-treatment	0.095 ± 0.030	0.301 ± 0.091	**<0.001**
p value vs. baseline	0.892	**<0.001**	
ΔC10	-0.002 ± 0.034	0.139 ± 0.074	**<0.001**
C4DC (nmol/L)			
Baseline	0.395 ± 0.125	0.367 ± 0.077	0.515
Post-treatment	0.429 ± 0.153	0.538 ± 0.105	0.053
p value vs. baseline	0.565	**<0.001**	
ΔC4DC	0.033 ± 0.059	0.170 ± 0.067	**<0.001**
C5DC (nmol/L)			
Baseline	0.574 ± 0.484	0.546 ± 0.169	0.851
Post-treatment	0.717 ± 0.566	1.203 ± 0.340	**0.018**
p value vs. baseline	0.514	**<0.001**	
ΔC5DC	0.143 ± 0.135	0.657 ± 0.256	**<0.001**
C12 (nmol/L)			
Baseline	0.070 ± 0.025	0.087 ± 0.021	0.094
Post-treatment	0.085 ± 0.036	0.129 ± 0.030	**0.003**
p value vs. baseline	0.257	**<0.001**	
ΔC12	0.015 ± 0.016	0.043 ± 0.023	**0.002**
C14:1 (nmol/L)			
Baseline	0.046 ± 0.013	0.065 ± 0.018	**0.006**
Post-treatment	0.050 ± 0.020	0.096 ± 0.026	**<0.001**
p value vs. baseline	0.583	**0.003**	
ΔC14:1	0.004 ± 0.016	0.031 ± 0.023	**0.003**
C14 (nmol/L)			
Baseline	0.089 ± 0.046	0.102 ± 0.048	0.496
Post-treatment	0.125 ± 0.082	0.161 ± 0.076	0.280
p value vs. baseline	0.196	**0.033**	

ΔC14	0.036 ± 0.051	0.059 ± 0.048	0.281
C14-OH (nmol/L)			
Baseline	0.050 ± 0.006	0.053 ± 0.027	0.752
Post-treatment	0.067 ± 0.049	0.105 ± 0.050	0.070
p value vs. baseline	0.295	**0.005**	
ΔC14-OH	0.017 ± 0.036	0.052 ± 0.046	**0.049**
C16 (nmol/L)			
Baseline	0.076 ± 0.026	0.082 ± 0.019	0.536
Post-treatment	0.079 ± 0.026	0.149 ± 0.035	**<0.001**
p value vs. baseline	0.768	**<0.001**	
ΔC16	0.003 ± 0.020	0.067 ± 0.026	**<0.001**
C16:1-OH (nmol/L)			
Baseline	0.008 ± 0.003	0.010 ± 0.002	0.090
Post-treatment	0.009 ± 0.004	0.016 ± 0.005	**<0.001**
p value vs. baseline	0.470	**<0.001**	
ΔC16:1-OH	0.001 ± 0.003	0.006± 0.003	**0.001**
C16-OH (nmol/L)			
Baseline	0.010 ± 0.003	0.010 ± 0.003	0.601
Post-treatment	0.011 ± 0.006	0.018 ± 0.005	**0.003**
p value vs. baseline	0.544	**<0.001**	
ΔC16-OH	0.001 ± 0.003	0.008 ± 0.005	**<0.001**
C18:1 (nmol/L)			
Baseline	0.067 ± 0.045	0.079 ± 0.028	0.445
Post-treatment	0.076 ± 0.032	0.150 ± 0.059	**<0.001**
p value vs. baseline	0.595	**0.001**	
ΔC18:1	0.009 ± 0.033	0.071 ± 0.044	**<0.001**
C18 (nmol/L)			
Baseline	0.025 ± 0.013	0.029 ± 0.006	0.344
Post-treatment	0.027 ± 0.008	0.053 ± 0.017	**<0.001**
p value vs. baseline	0.659	**<0.001**	
ΔC18	0.002 ± 0.009	0.024 ± 0.013	**<0.001**
C18:1-OH (nmol/L)			
Baseline	0.004 ± 0.001	0.006 ± 0.001	**0.015**
Post-treatment	0.005 ± 0.003	0.008 ± 0.002	**0.001**
p value vs. baseline	0.443	**0.004**	
ΔC18:1-OH	0.001 ± 0.002	0.002 ± 0.002	**0.024**
C18-OH (nmol/L)			
Baseline	0.004 ± 0.002	0.005 ± 0.001	0.196
Post-treatment	0.005 ± 0.003	0.008 ± 0.002	**0.001**
p value vs. baseline	0.365	**<0.001**	

ΔC18-OH	0.001 ± 0.002	0.003 ± 0.002	**0.002**
Short-chain Acyl-C (C2+C3) (nmol/L)			
Baseline	9.346 ± 1.788	9.381 ± 2.230	0.966
Post-treatment	8.505 ± 1.277	36.489 ± 14.007	**<0.001**
p value vs. baseline	0.198	**<0.001**	
ΔShort-chain Acyl-C	-0.841 ± 1.332	27.108 ± 13.403	**<0.001**
Middle + Long-chain Acyl-C (C4 to C18-OH) (nmol/L)			
Baseline	2.617 ± 0.952	3.746 ± 3.237	0.259
Post-treatment	2.910 ± 1.072	6.469 ± 1.151	**<0.001**
p value vs. baseline	0.487	**0.012**	
ΔMiddle -Long-chain Acyl-C	0.293 ± 0.465	2.723 ± 3.144	**0.015**
The ratio of short / middle-long chain Acyl-C			
Baseline	3.829 ± 1.064	3.202 ± 1.096	0.169
Post-treatment	3.145 ± 0.861	5.589 ± 1.523	**<0.001**
p value vs. baseline	0.097	**<0.001**	
ΔS / M+L ratio	-0.685 ± 0.576	2.387 ± 1.238	**<0.001**

Values are shown as mean ± SD or range.

Acyl-C=acylcarnitine.

Supplementary Table 7. Primer sequences for real-time PCR

Targeted gene	Forward	Reverse
Il6	TTCCATCCAGTTGCCTTCTT	TTTCTCATTTCCACGATTTCC
Tnfa	GCCTCTTCTCATTCCTGCTT	GATCTGAGTGTGAGGGTCTGG
Ccl2	CCACTCACCTGCTGCTACTC	TGTCTGGACCCATTCCTTCT
Il18	ACAACTTTGGCCGACTTCAC	GGGTCACAGCCAGTCCTCT
Il1b	GCATCCAGCTTCAAATCTCAC	GGTGCTCATGTCCTCATCCT
Acta2	AGGGCTGGAGAATTGGATCT	CCAGCAAAGGTCAGAGAAGG
Pdgfrb	CTGTCCGTGTTATGGCTCCT	GGGACATCTGTTCCCACATC
Timp2	GTTTTGCAATGCAGATGTAG	ATGTCGAGAAACTCCTGCTT
Tmlhe	CTGTGCCTTACGACGTTGTC	AGTTGACGGTAGCCAGTGAA
18s ribosome RNA	GACTCAACACGGGAAACCTC	AACCAGACAAATCGCTCCAC

Il, interleukin; Tnf, tumor necrotic factor; Ccl, C-C motif chemokine ligand 2; Acta2, actin alpha 2, smooth muscle; Pdgfrb, platelet derived growth factor receptor beta; Timp2, tissue inhibitor of metalloproteinases 2; Tmlhe, trimethyllysine hydroxylase, epsilon.
