# Supplementary material for: Involvement of impaired carnitine-induced fatty acid oxidation in experimental and human diabetic kidney disease
Source: JCI Insight. 2025 May 22;10(13):e179362. doi: 10.1172/jci.insight.179362 (PMC12288908; doi:10.1172/jci.insight.179362)
Supplement: Unedited blot and gel images [file jciinsight-10-179362-s041.pdf]

Involvement of impaired carnitine-induced fatty acid oxidation in experimental and human diabetic kidney disease

Sakuya Ito, Kensei Taguchi*, Goh Kodama, Saori Kubo, Tomofumi Moriyama, Yuya Yamashita, Yunosuke Yokota, Yosuke Nakayama, Yusuke Kaida, Masami Shinohara, Kyoko Tashiro, Keisuke Ohta, Sho-ichi Yamagishi, and Kei Fukami

Figure 4A

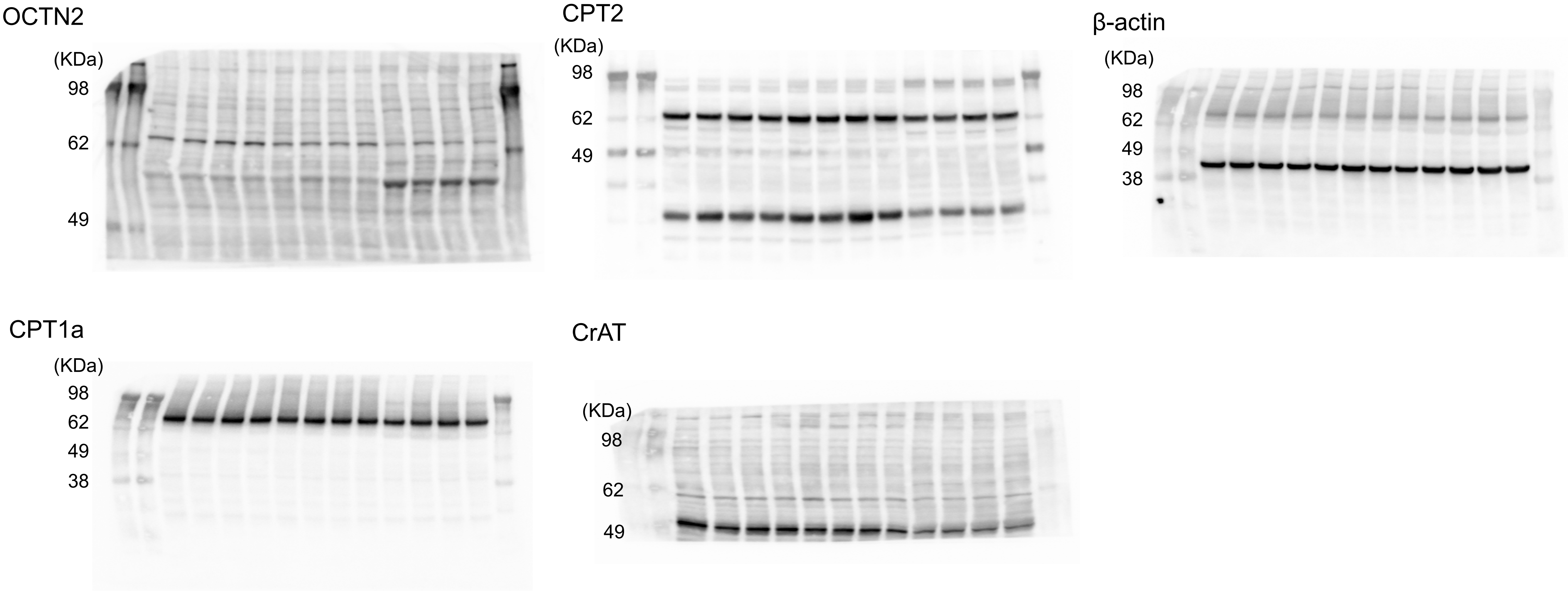

Figure 4F

p-AMPK

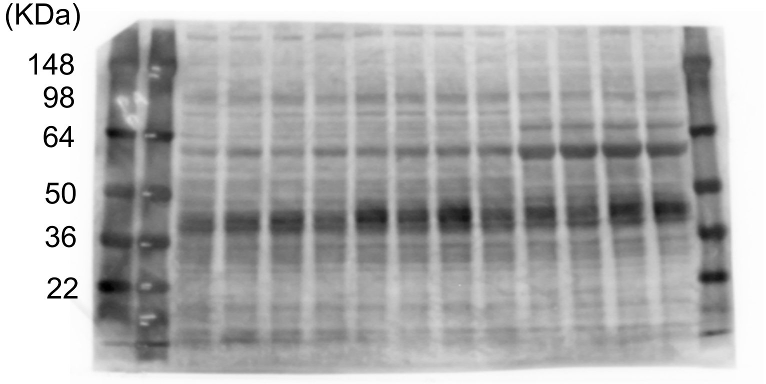

PGC-1α

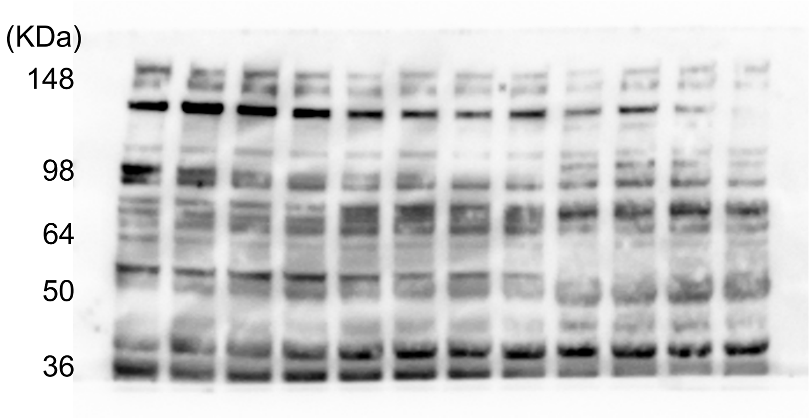

AMPK

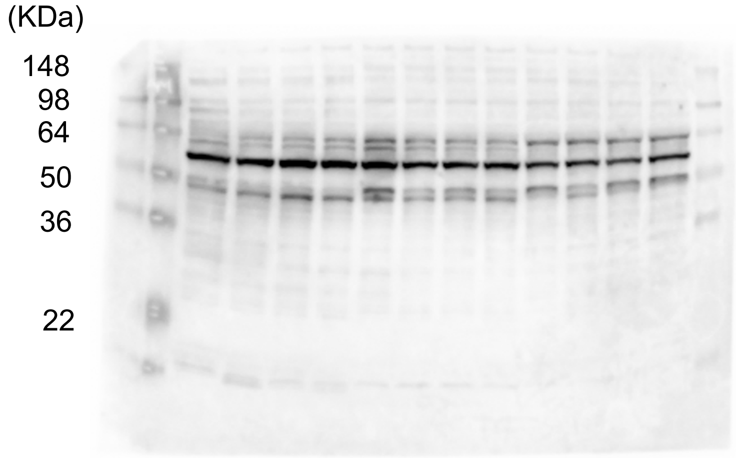

β-actin

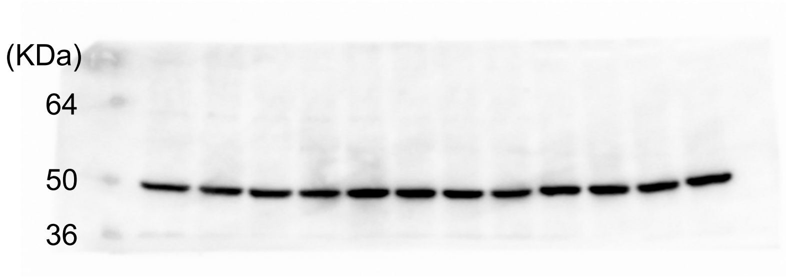

Figure 5C

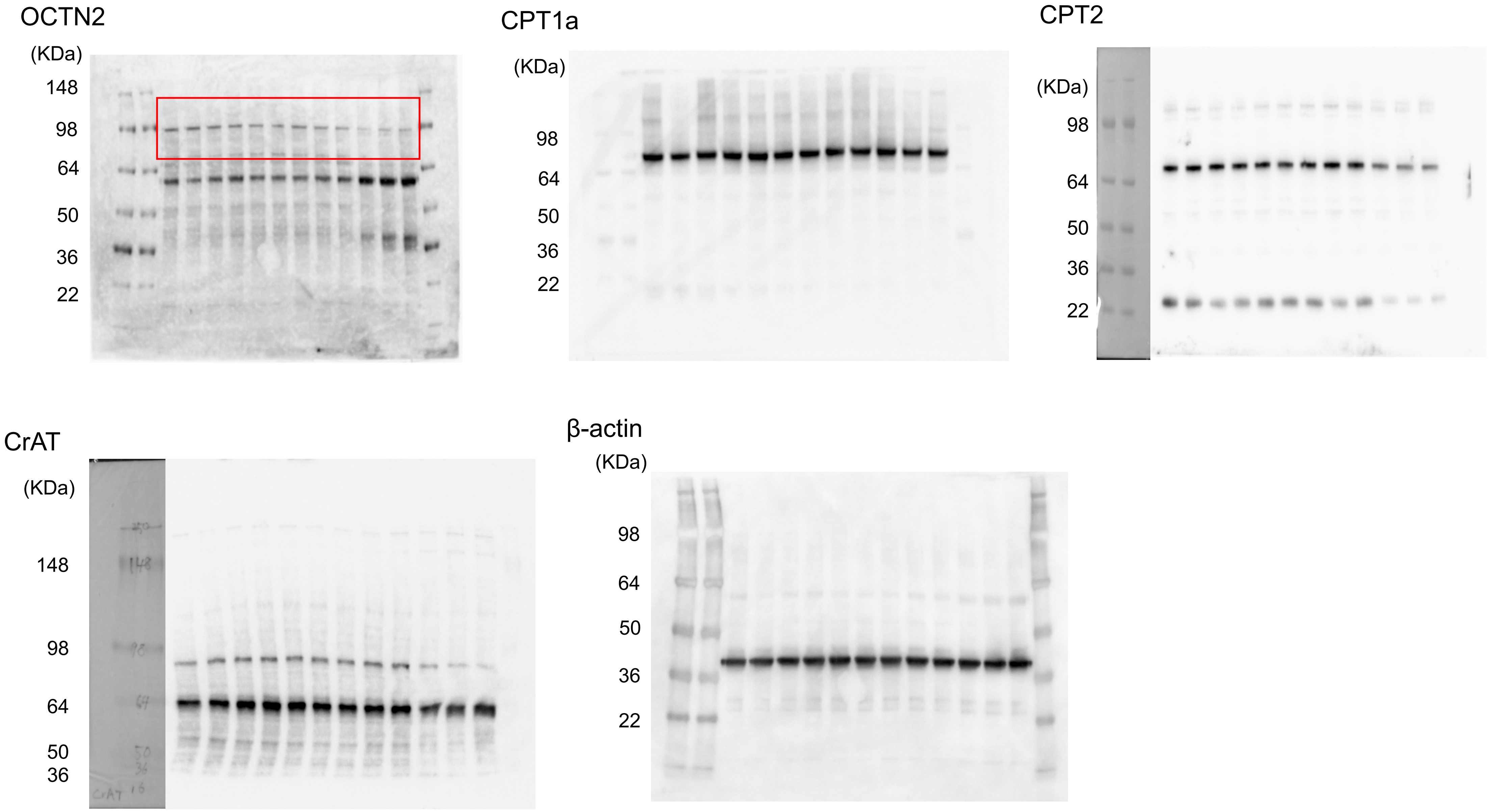

OCTN2
(KDa)
148
98
64
50
36
22
CPT1a
(KDa)
98
64
50
36
22
CPT2
(KDa)
98
64
50
36
22
CrAT
(KDa)
148
98
64
50
36
β-actin
(KDa)
98
64
50
36
22

Figure 7A

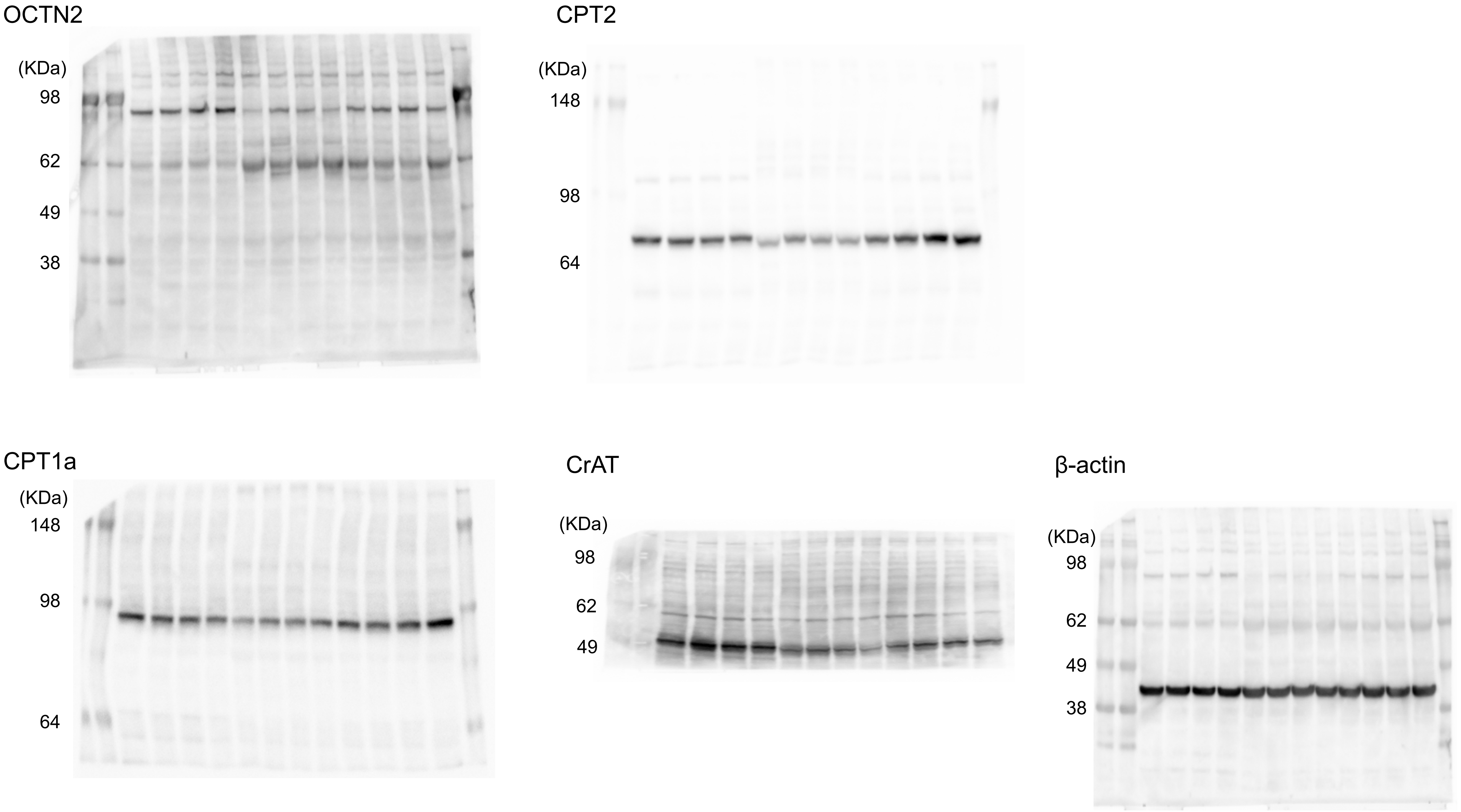
OCTN2
(KDa)
98
62
49
38
CPT2
(KDa)
148
98
64
CPT1a
(KDa)
148
98
64
CrAT
(KDa)
98
62
49
β-actin
(KDa)
98
62
49
38

Figure 7D

p-AMPK

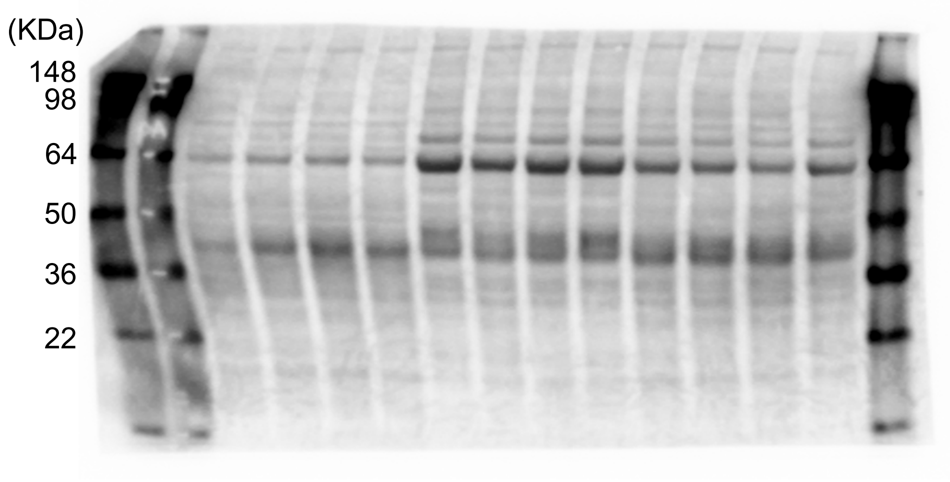

β-actin

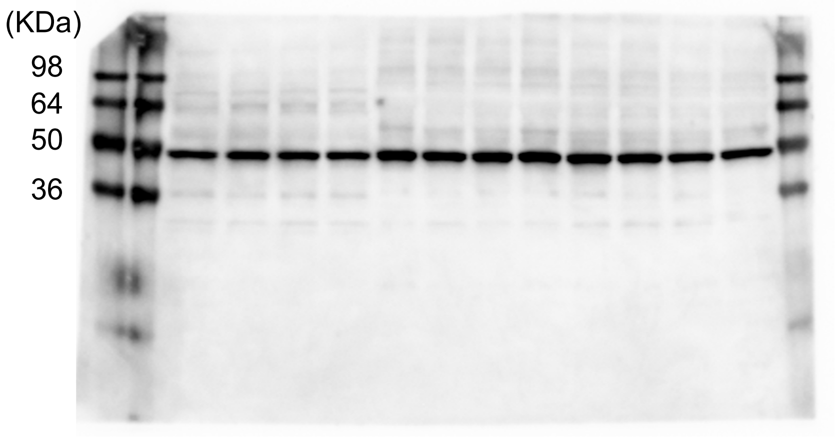

AMPK

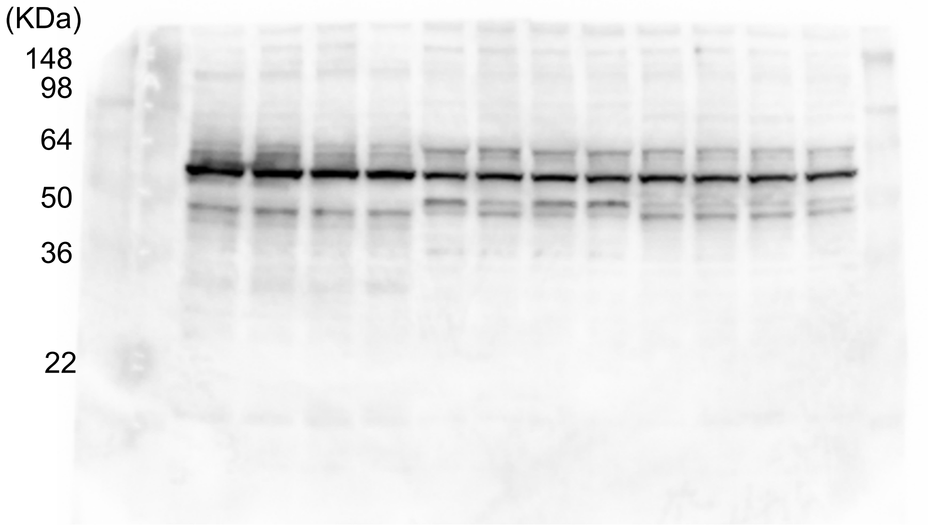

Figure 7F

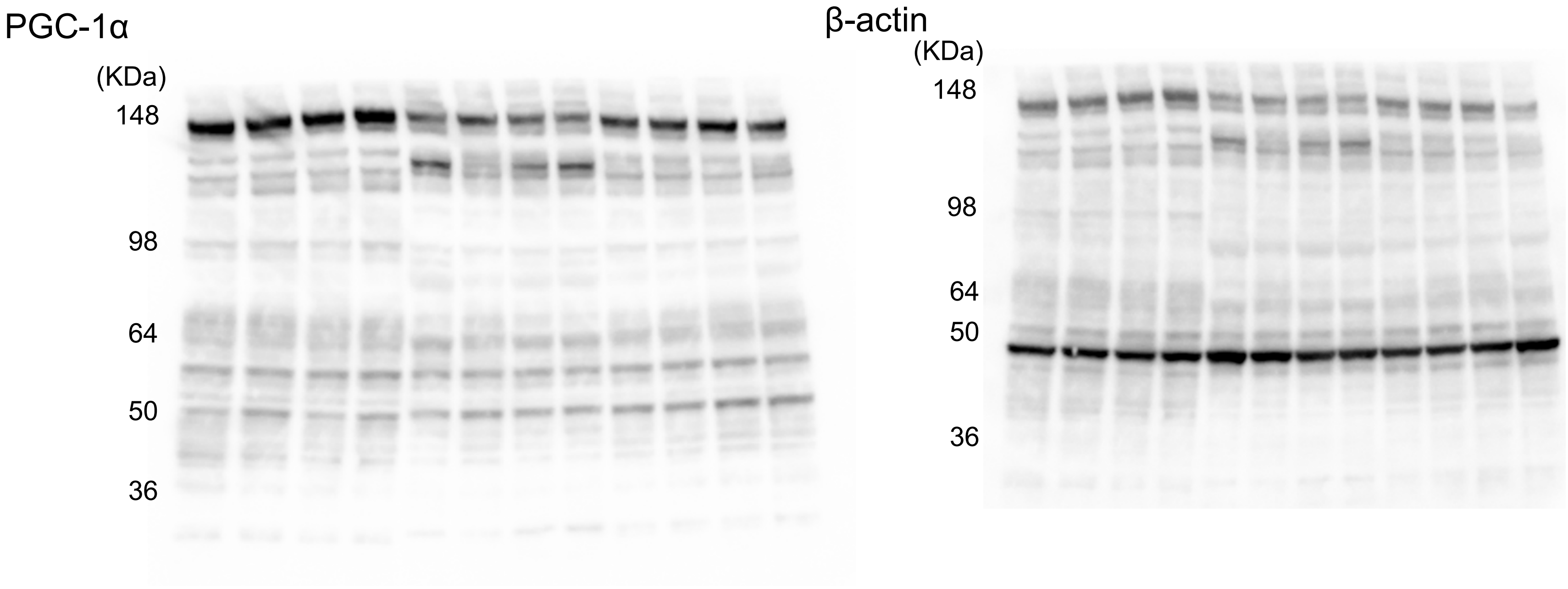

Figure 8D

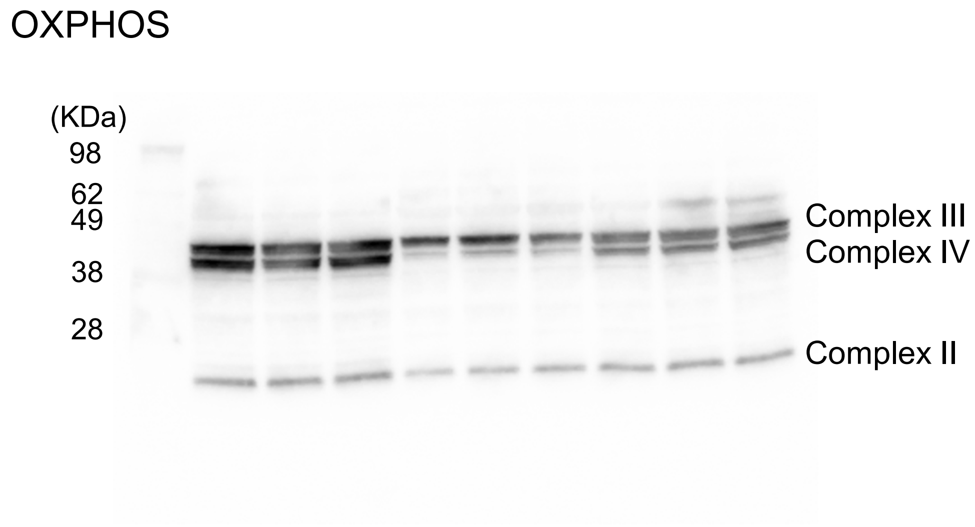

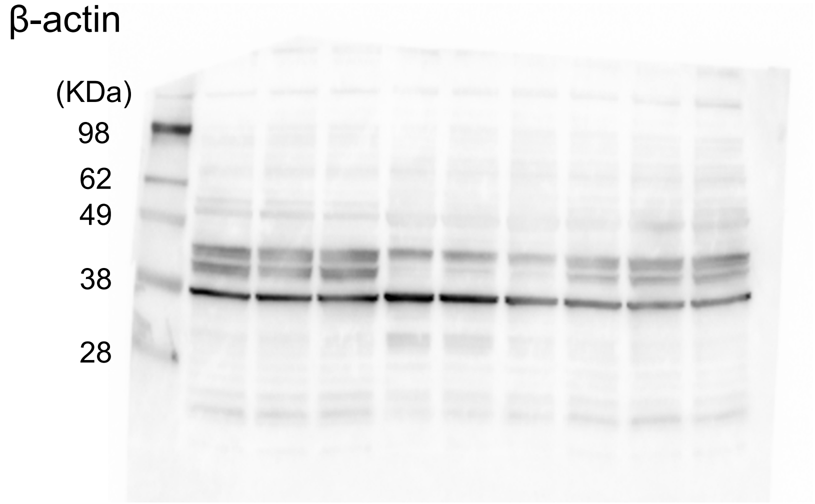

Figure 8F

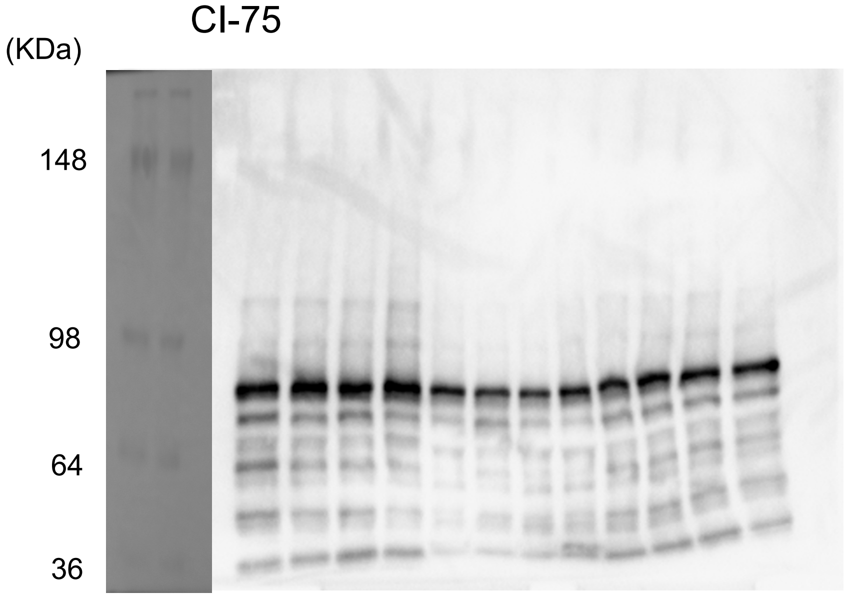

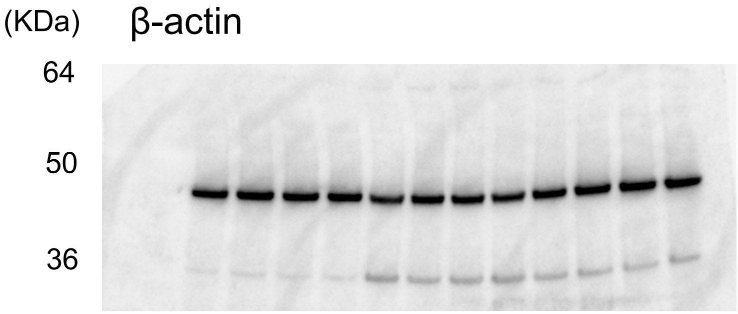

Figure 8K

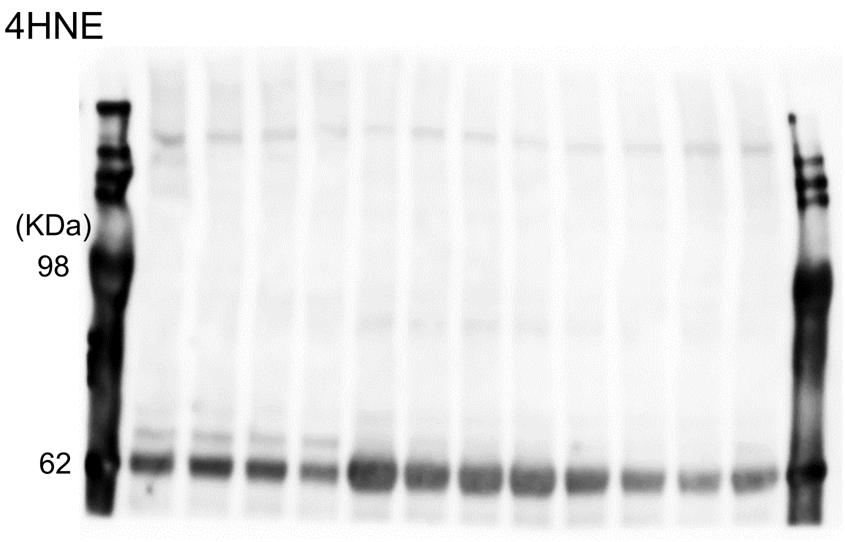

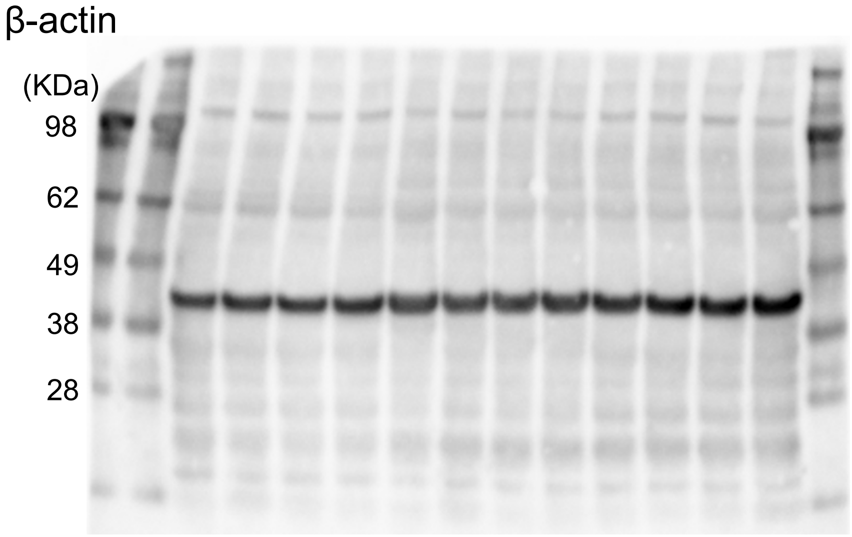

Figure 9D

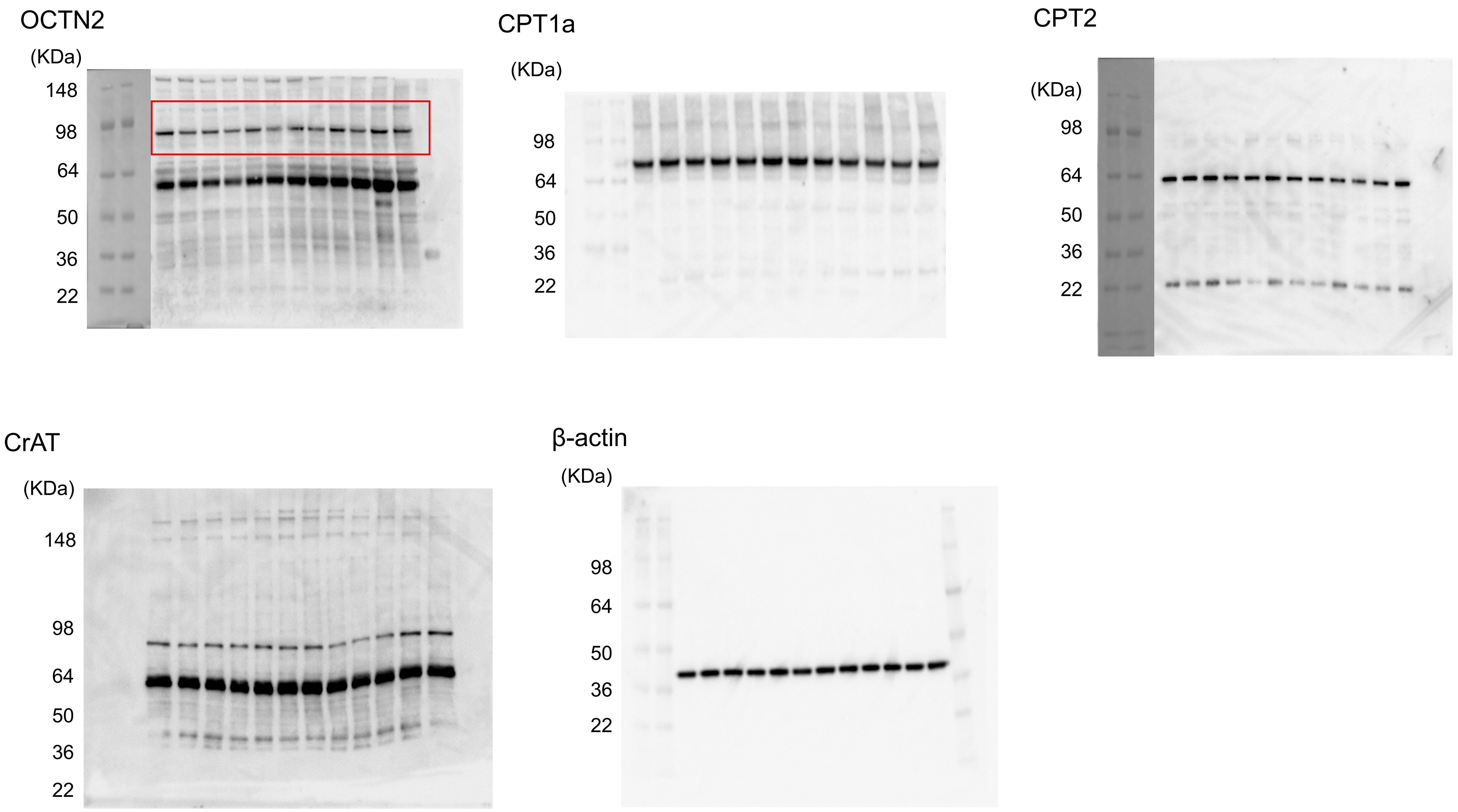

OCTN2
(KDa)
148
98
64
50
36
22
CPT1a
(KDa)
98
64
50
36
22
CPT2
(KDa)
98
64
50
36
22
CrAT
(KDa)
148
98
64
50
36
22
β-actin
(KDa)
98
64
50
36
22
